# *C. elegans* as a test system to study relevant compounds that contribute to the specific health-related effects of different cannabis varieties

**DOI:** 10.1186/s42238-022-00162-9

**Published:** 2022-10-03

**Authors:** Monique van Es-Remers, Jesus Arellano Spadaro, Eefje Poppelaars, Hye Kyong Kim, Marieke van Haaster, Marcel de Wit, Eva ILiopoulou, Marjolein Wildwater, Henrie Korthout

**Affiliations:** 1 Vivaltes B.V., Bunnik, Regulierenring 9, 3981 LA Bunnik the Netherlands; 2 Fytagoras B.V., Sylviusweg 72, 2333 BE Leiden, the Netherlands; 3 Maripham B.V., Nieuw-Mathenesserstraat 33, 3029 AV Rotterdam, the Netherlands

**Keywords:** *C. elegans*, Medicinal cannabis, Entourage effects, Appetite, Motility, Body oscillation, Nervous system, Memory, Endurance performance

## Abstract

**Background:**

The medicinal effects of cannabis varieties on the market cannot be explained solely by the presence of the major cannabinoids Δ^9^-tetrahydrocannabinol (THC) and cannabidiol (CBD). Evidence for putative entourage effects caused by other compounds present in cannabis is hard to obtain due to the subjective nature of patient experience data. *Caenorhabditis elegans* (*C. elegans*) is an objective test system to identify cannabis compounds involved in claimed health and entourage effects.

**Methods:**

From a medicinal cannabis breeding program by MariPharm BV, the Netherlands a set of 12 varieties were selected both THC rich varieties as well as CBD rich varieties. A consecutive extraction process was applied resulting in a non-polar (cannabinoid-rich) and polar (cannabinoid-poor) extract of each variety. The test model *C. elegans* was exposed to these extracts in a broad set of bioassays for appetite control, body oscillation, motility, and nervous system function.

**Results:**

Exposing *C. elegans* to extracts with a high concentration of cannabinoids (> 1 μg/mL) reduces the life span of *C. elegans* dramatically. Exposing the nematodes to the low-cannabinoid (< 0.005 μg/mL) polar extracts, however, resulted in significant effects with respect to appetite control, body oscillation, motility, and nervous system-related functions in a dose-dependent and variety-dependent manner.

**Discussion:**

*C. elegans* is a small, transparent organism with a complete nervous system, behavior and is due to its genetic robustness and short life cycle highly suitable to unravel entourage effects of Cannabis compounds. Although *C. elegans* lacks an obvious CB1 and CB2 receptor it has orthologs of Serotonin and Vanilloid receptor which are also involved in (endo)cannabinoid signaling.

**Conclusion:**

By using *C. elegans*, we were able to objectively distinguish different effects of different varieties despite the cannabinoid content. *C. elegans* seems a useful test system for studying entourage effects, for targeted medicinal cannabis breeding programs and product development.

**Supplementary Information:**

The online version contains supplementary material available at 10.1186/s42238-022-00162-9.

## Background

The medicinal use of cannabis has a long history of several thousands of years and has been reported in ancient Ayurvedic and Chinese Medicine (Pisanti and Bifulco 2018). Although cannabis was introduced in modern Western medicine in the nineteenth century, the real breakthrough came with the discovery of cannabinoids (Mechoulam and Gaoni 1967) and the discovery of the endocannabinoid systems with its receptors (Pertwee 1993; Mackie 2008). From that moment on, research activities and clinical applications of medicinal cannabis were mainly focused on the major cannabinoid Δ^9^-tetrahydrocannabinol (THC), and later on cannabidiol (CBD). As a consequence, purified and synthetic THC was produced by the pharmaceutical industry for a broad range of medicinal applications. Interestingly, patients’ experiences indicated that extracts of cannabis could in certain circumstances be more beneficial compared to purified or synthetic cannabinoids. For example, for multiple sclerosis, it was reported that cannabis extracts which contained a full spectrum of metabolites like cannabinoids, terpenoids, and flavonoids, were more beneficial in clinical applications compared to purified cannabinoids like THC/CBD, or pharmaceutical products thereof (Maayah et al. 2020). The first scientific reports on the putative entourage effect of cannabis, in which other compounds like terpenoids and flavonoids were involved, already appeared some decades ago (McPartland and Russo 2001; Russo 2011). However scientific studies on entourage effects of cannabis have been focused mainly on the role of minor cannabinoids and terpenoids (Koltai and Namdar 2020) but there are no clear indications reported on the role of other compounds like flavonoids or other molecules.

Cannabinoids, terpenoids, and flavonoids all belong to the class of secondary metabolites, and the content of these compounds in a cannabis plant strongly depends on both the genotype as well as on growing- and processing conditions of the plant. These secondary metabolites in cannabis dictate flavor, smell, and perception. A recent study among 100,000 recreational users and more than 1200 cannabis varieties revealed a correlation between the metabolite content and consumers’ perception (de la Fuente et al. 2020). In addition, secondary metabolites are responsible for the health-related properties. The fact that patients have a strong preference for a typical variety for a typical medicinal indication might furthermore be due to differences in composition of secondary metabolites. Objective scientific data that supports the causal relation between the cannabis genotype/chemotype with their medicinal effects is, however, still very limited.

The nematode *Caenorhabditis elegans* (*C. elegans*) is a transparent organism that has been successfully used as a key model system in human disease gene discovery (Apfeld and Alper 2018). The organism contains a digestive-, reproductive, and nervous system, as well as musculature, and it exhibits complex behaviours. Currently, the use of *C. elegans* as a model system for medicinal cannabis studies is limited. This might be due to the fact that homologs of the cannabinoid receptors CB1 and CB2 are missing in *C. elegans*. On the other hand, it was recently shown that the endocannabinoids 2-arachidonoylglycerol (2-AG) and anandamide (AEA) exert multiple behavioral effects in *C. elegans* via the cannabinoid-like receptor NPR-1 (Oakes et al. 2017). In addition, the same research group showed that another endocannabinoid signalling pathway was present in *C. elegans*; which was independent on the cannabinoid-like receptor pathway but acts via transient receptor potential vanilloid 1 (TRPV1) and TRPN-like channels (Oakes et al. 2019). In humans, it is also shown that (endo)cannabinoid signalling does not only act via CB1 and CB2 receptors but that also other targets are involved, such as the vanilloid-type TRP channels and the serotonin receptor 5-HT_1A_ (Di Marzo and Piscitelli 2015; Pazos et al. 2013; de Almeida and Devi 2020). Interestingly, it has been postulated that putative entourage effects, in case of terpenoids, are not mediated by the CB1 and/or CB2 receptor (Santiago et al. 2019). Taken together, these findings enable the use of *C. elegans* as an objective model system to test correlations between the cannabis genotype and chemotype on the one hand, as well as their medicinal and/or behavioral effect on the other hand.

In this study, a set of 12 different cannabis varieties was tested on a broad set of *C. elegans*-based bioassays, representing several indications in which cannabis is therapeutically used, e.g., appetite control, motility, body oscillation, and nervous system functioning.

## Methods

### Plant materials

In this study, a set of 12 different cannabis varieties were analyzed, which were established by a medicinal-grade breeding program of MariPharm started in 1994 in Rotterdam, the Netherlands. This set of 12 varieties was selected based on the beneficial effects in relation to appetite control, muscle relaxation and neurodegenerative diseases as reported by cannabis users. Three of the varieties MGC 1013, MGC 1074, and MGC 1122 were CBD-rich varieties; nine of them MGC 1003, MGC 1007, MGC 1009, MGC 1010, MGC 1027, MGC 1046, MGC 1101, and MGC 1106 were THC-rich varieties. Plants were registered at Community Plant Variety Office (CPVO), Angers, France.

### Chemicals

Chemicals that were used are: 1-Butanol, ≥ 99% (Casnr. 71-36-3, Sigma-Aldrich, Schnelldorf, Germany, B7906); Benzaldehyde, ≥ 99% (Casnr. 100-52-7, Sigma-Aldrich, Schnelldorf, Germany, B1334); 1-Octanol anhydrous, ≥ 99% (Casnr. 111-87-5, Sigma-Aldrich, Schnelldorf, Germany, 297887); *n*-Hexane, ≥ 95% (Casnr. 110-54-3, Sigma-Aldrich, Schnelldorf, Germany, 270504); Ethanol, 99.8% (Casnr. 64-17-5, Antonides, Vollenhove, The Netherlands, 10152721); DMSO, 100% (Casnr. 67-68-5, Antonides, Vollenhove, The Netherlands, I-12.779-1000); and Tween 20 (Casnr.9005-64-5, Sigma-Aldrich, Schnelldorf, Germany, P9416). Pure cannabinoid compounds were obtained from MariPharm.

### Nematode growth medium agar (NGM)

For 1 L NGM agar, 17 g of agar (CASnr. 9002-18-0?; formedium; artnr. AGA03), 2.5 g of peptone (CASnr. 73049-73-7; formedium; artnr. PEP03), and 3.0 g of NaCl (sodium chloride; CASnr. 7647-14-5; e.g., Sigma, artnr. S5886) were transferred into an autoclavable 1 L bottle, where 975 mL of Milli-Q was added. The bottle was autoclaved for 20 min at 120 °C and let cool to approx. 55 °C. Then, 25 mL of (sterile) potassium phosphate buffer (see “Potassium phosphate buffer” section), 1 mL of (sterile) 1 M MgSO_4_ (120 mg/mL) (magnesium sulfate; CASnr. 7487-88-9; e.g., Sigma, artnr. M2643), 1 mL of (sterile) 1 M CaCl_2_ (147 mg/mL) (calcium chloride·2H_2_O; CASnr. 10035-04-8; e.g., Sigma; artnr. C3881), and 1 mL of (sterile) cholesterol (5 mg/mL) (CASnr. 57-88-5; e.g., Sigma; artnr. C8667) were added and mixed well. The bottle was left for 1 min to prevent air bubbles, before pouring or pipetting the resulting NGM agar into petri dishes or into well plates.

### Potassium phosphate buffer

To create the potassium phosphate buffer, 35.66 g of K_2_HPO_4_ * 3H_2_O (potassium dibasic phosphate trihydrate; CASnr. 16788-57-1; Sigma P5504) and 108.2 g of KH_2_PO_4_ (Potassium phosphate monobasic; CASnr. 7778-77-0; Sigma P5655) were dissolved in Milli-Q up to 1 L (pH 6.0). The buffer was autoclaved at 120 °C for 20 min, after which the pH was checked again.

### M9 buffer

To create the M9 buffer, 3.0 g of KH_2_PO_4_ (potassium phosphate; CASnr. 7778-77-0; e.g., Sigma, P5655), 7.5 g of Na_2_HPO_4_ * 2 H_2_O (sodium phosphate dihydrate; CASnr. 10028-24-7; e.g., Sigma, 30412), and 5.0 g of NaCl (sodium chloride; CASnr. 7647-14-5; e.g., Sigma, S5886) were transferred into an autoclavable 1 L bottle and filled until 1000 mL with Milli-Q. The bottle was autoclaved for 20 min at 120 °C, and let cool to at least 55 °C. In a sterile environment, 1 mL of 1 M MgSO_4_ (magnesium sulphate; CASnr. 7487-88-9; e.g., Sigma, M2643) was added and mixed well.

### M9 tween buffer

To prepare a 10% Tween® 20 (CASnr. 9005-64-5; e.g., Sigma, P9416) solution, Tween-20 was diluted ten times with Milli-Q, and filter sterilized using a sterile 0.22 μm filter. Two hundred fifty microliters of the 10% Tween 20 solution was added to 50 mL of M9 buffer (see “M9 buffer” section) and mixed well to create a final concentration of 0.05% Tween 20.

### *C. elegans* strains and culture conditions

*Caenorhabditis elegans* (*C. elegans*) N2 hermaphrodites (wild-type) were obtained from the *C. elegans* Genome Center (CGC). The strain was maintained on agar nematode growth medium (NGM) in dark incubators at 16 °C (unless otherwise specified). Nematodes were incubated at 16 °C to obtain a population for experimental testing. Compound stock was prepared by dissolving dried extracts in Dimethyl sulfoxide (DMSO), ethanol, or *n*-hexane (dependent on experiment) in Eppendorf tubes, a ratio of 1:1 was used in comparison to the original extraction volume. Compound solution and MQ (Milli-Q purified water) were added to Falcon tubes, to which two times NGM agar (cooled to 65 °C) was added to a final one times concentration. The solution was mixed well and 7 mL of the agar was pipetted into 6-cm plastic petri dishes (unless stated otherwise). Plates were stored overnight at room temperature in a flow cabinet. Plates were seeded with 100 μL three times concentrated *Escherichia coli* strain OP50 bacteria (unless stated otherwise) that were grown in liquid culture to a density of OD_600_ = 0.700–1.000. The plates were stored overnight at room temperature in a flow cabinet. Nematodes were age-synchronized by hypochlorite bleaching and incubated between 16 and 24 h in M9 with 0.05% Tween 20 at 20 °C without food to obtain larvae in L1 arrest. The concentration of L1 larvae was calculated and the appropriate volume of L1 nematodes needed for an experiment were pipetted to the compound plates.

### *C. elegans* behavioral scoring

Different observers performed the cross-validated assays according to scientific literature adjusted into Standard Operational Procedures. Tests were observed and analyzed using blinded and coded sample names to prevent subconscious bias in the analysis/scoring.

### Life span assay

To measure life span with cannabis treatment, 100 L1 nematodes were grown on 6-cm agar plates that contained cannabis extract. The number of alive nematodes was determined every one, two, or three days until all nematodes were dead. All the alive nematodes were transferred to freshly made agar plates that contained cannabis extract every 1, 2, or 3 days.

### Pharyngeal pumping assay

The pharyngeal pumping activity in presence or absence of cannabis extracts was measured as described by Raizen et al. (Raizen et al. 2005-2018). Briefly, pharyngeal pumping (neuromuscular contraction in the presence of food) was assessed after 3 days of incubation at 20 °C by counting the number of contractions within 15 s. At least 15 nematodes were scored for each condition.

### Oscillations assay

To measure motility, the number of body bends (oscillations) within a set time frame were counted. Nematodes were individually transferred to new plates next to the bacterial food where they were allowed to acclimate for 30 s, the number of complete oscillations (composed of two body bends) was counted during 30 s. At least fifteen nematodes were scored for each condition per experiment.

### 1-Octanol assay

1-octanol reversal tests were performed as reported in the literature (Chao et al. 2004). 1-octanol is a repellent odor for *C. elegans* and nematodes respond to the presence of 1-octanol by moving away from it when placed at one body distance in front of the head of the nematode. Two hundred L1 nematodes were grown for 3 days at 20 °C on 6-cm agar plates that contained vehicle (control) or cannabis extract. Nematodes were transferred to fresh NGM plates without food and acclimated for 10 min. An eyelash pick dipped in 30% 1-octanol (solved in ethanol) was positioned at body-length distance in front of a moving nematode. The number of seconds until nematodes completed a full-sinusoidal backward movement was scored. A total of 30 nematodes were tested per condition. Octanol avoidance behavior in *C. elegans* is proven to be linked to dopamine signalling (Baidya et al. 2014). Forty microliters of a freshly prepared 1 M dopamine solution in Milli-Q ultrapure water (MQ) was placed on an empty 6-cm NGM plate and allowed to dry for 10 min in the dark. Forty microliters of MQ was used as a control plate. Nematodes were transferred to these NGM plates and acclimated for 10 min. An eyelash pick dipped in 30% 1-octanol (solved in ethanol) was positioned at one body-length distance in front of a moving nematode. The number of seconds until nematodes completed a full-sinusoidal backward movement was scored.

### Endurance assay

Endurance assays were performed as reported in literature (Laranjeiro et al. 2017). L1 nematodes were grown for three days at 20 °C on 6-cm agar plates that contained vehicle (control) or cannabis extract. Nematodes were washed of the NGM plates and put to swim for 120 min in 2 ml M9-tween buffer. A negative control condition with crawling nematodes on an empty NGM plate was used for every assay, as well as a non-treated swimming condition. After 120 min of exercise, 10 μL of the M9-tween buffer containing the nematodes was put on a seeded NGM plate. After exactly 5 min, the distance crawled by the nematodes was measured. For every condition two droplets of 10 μL were measured, with approximately fifteen nematodes per droplet. The assay starts with the non-swimming (crawling) control condition and ends again with the non-swimming (crawling) condition, which were compared to verify a consistent quality of the assay over the course of the experiment.

### Adverse memory assay

Adverse memory assays were performed as reported in literature (Eliezer et al. 2019). L1 nematodes were grown for three days at 20 °C on 10-cm agar plates that contained vehicle (control) or cannabis extract. Nematodes were washed of the plates with M9-Tween buffer and washed two more times with M9-Tween buffer. The pellet of nematodes was transferred to starvation plates by distributing the nematodes over two labeled empty 10 cm NGM plates per condition. When plates were dry, nine droplets of 5 μL of butanone 10% for the conditioned nematodes and nine droplets of 5 μL of MQ for the unconditioned nematodes were pipetted on the lid of the plate. Plates were left at room temperature for 24 h. Nematodes were washed off the plates with M9-Tween buffer, washed two times with M9-Tween buffer after which nematodes were transferred to recovery plates for 4 h before the assay was initiated. The assay was executed using two plates per condition which were divided in 4 quadrants (see Additional file S1). 1.5 μL sodium azide (1 M) was pipetted in E and B duplicate quadrants, 5 μL ethanol (1%) in both E quadrants, and 5 μL butanone (1%) in both B quadrants. Six microliters of *C. elegans* pellet was pipetted onto the middle of the assay plate. Nematodes were left on the plates for 1 h at room temperature after which the plate was scored by determining the number (*n*) of nematodes in the B quadrants, E quadrants, and the remaining nematodes in the plate with a cell counter clicker. The chemotaxis index (CI) was calculated as:

$$CI=\frac{n\ nematodes\ in\ B-n\ nematodes\ in\ E}{total\ n\ of\ nematodes}$$

### Consecutive extraction

For each cultivar, three flower tops were each dried and ground to an ultra-fine powder. Each powder was first extracted by a Pressurized Solvent Extractor (PSE) E-916 (BÜCHI Labortechnik AG, Flawil, Switzerland) based on the method described by Bayona et al. (Bayona et al. 2018). Briefly, 1000 mg of dried and homogenized flower top powder, mixed with 5 mL fat-free quartz sand (0.3–0.9 mm, BÜCHI Labortechnik AG, Flawil, Switzerland), was first extracted with 100% *n*-hexane in 10 mL stainless steel extraction cells at 45 ^o^C and 100 bar. Two cycles (heat-up time of 1 min hold time of 1 min for cycle 1 and 2 min for cycle 2, and discharge time of 2 min) were performed to finally obtain the non-polar fraction (NPF). The remaining material in the extraction cells was then extracted with 70% ethanol under the same conditions to obtain the polar fraction (PF). The *n*-hexane extracts were divided into two equal parts, and the *n*-hexane was removed by evaporation under nitrogen. The first part of the non-polar fraction (NPF) was left untreated, while the second part was heated at 143 ^o^C for 7 min in a sand bath to allow decarboxylation of the acidic cannabinoids, to obtain the decarboxylated non-polar fraction (NPF_dec_). All obtained fractions were dried under nitrogen and resolved in ethanol prior to exposure experiments with *C. elegans*.

### NMR analysis

Extracts were transferred to 2 mL Eppendorf tubes and dissolved in 1 mL CH_3_OH-d_4_ (Cambridge Isotope Laboratories, MA, USA) with hexamethyldisiloxane (HMDSO) as an internal standard. The extracts were vortexed vigorously and sonicated for 5 min. After centrifugation at 13,000 rpm for 10 min, 300 μL of the supernatant were transferred to 3-mm NMR tubes for ^1^H-NMR measurement. ^1^H-NMR analysis was performed using the method described by López-Gresa et al. (López-Gresa et al. 2012). Briefly, ^1^H-NMR spectra were recorded at 25 °C on a 600-MHz Bruker AV 600 spectrometer equipped with a TCI cryo-probe. CH_3_OH-d_4_ was used for the internal lock. Each ^1^H-NMR spectrum consisted of 64 scans requiring 5-min acquisition time. The experimental parameters were as follows: 0.25 Hz/point; pulse width (PW) = 30° (10.8 μs); and relaxation delay (RD) = 1.5 s. Free induction decay (FIDs) were Fourier transformed with Line Broadening (LB) = 0.3 Hz and the spectra were zero-filled to 32 K points. The resulting spectra were manually phased, baseline corrected, and calibrated to CH_3_OH-d_4_ at 3.30 ppm, using Topspin (version 3.5, Bruker). ^1^H-NMR spectra were automatically reduced to ASCII files using AMIX software (v. 3.7, Bruker Biospin). Bucket data was obtained by spectra integration at every 0.04 ppm interval from 0.40 to 10.00 ppm. The peak intensity of individual peaks was scaled to the total intensity of the buckets. The region of 4.7–4.9 ppm was excluded from the analysis because of the residual signal of water, as well as 3.28–3.34 ppm for residual methanol. Principal component analysis (PCA) was performed using MetaboAnalyst 5.0 (https://www.metaboanalyst.ca).

### HPLC analysis

Cannabinoids in different extracts were analyzed and quantified by means of HPLC. Samples were injected to the Agilent 1200 Series HPLC as following conditions. A C-18 column (Luna 5 μm, 150 x 4.6 mm) was used (Phenomenex). The mobile phase consisted of solvent A with water with 0.1% formic acid ranging from 70% till 10% in 27 min and solvent B with acetonitrile with 0.1% formic acid ranging from 30% till 90%. Injection volume was 5 μL, flow rate was 1.0 mL/min and column oven temperature was set to 30 °C. Detection was done at 280 nm by the DAD detector. Quantification was performed by using analytical standards.

### Statistical analysis

For statistical analysis, R version 4.1.2 (R Core Team 2022) with RStudio 1.4.1717 (RStudio Team Inc. 2021) was used. Differences between the vehicle control and experimental conditions within experiments were analyzed using analysis of variance tests (ANOVAs), and post-hoc pairwise testing was done using either *t* tests, Welch tests, or Mann-Whitney *U* tests (depending on assumption violations). Figures were plotted using R package *ggplot2* (Wickham 2016). Survival analysis was performed using R package *survival* 3.3.1 (Therneau 2022) and plotted using *survminer* 0.4.9 (Kassambara et al. 2021). Multiple comparisons correction was applied by using the false discovery rate (FDR) (Benjamini and Hochberg 1995). The significance level was set at FDR-corrected *p* < 0.05.

## Results

Breeding of different cannabis varieties has a long tradition worldwide. Next to an extended cannabis breeding program for recreational use, several programs have been started for medicinal use. From a medicinal-grade breeding program, a set of 12 varieties were selected based on the variance in genetic background, cannabinoid levels, and anecdotal health effects reported to MariPharm in the past 25 years. Among these 12 varieties are 3 CBD-rich varieties and 9 THC rich varieties.

### Exposure of *C. elegans* to cannabis extracts

In general, the content of cannabinoids in extracts from cannabis flowers is very high, as compared to other compounds like terpenoids and flavonoids. In humans it is known that cannabinoids have an extensive first pass metabolism by the liver after oral administration (Lucas et al. 2018). This will very likely result in a different ratio between cannabinoids and other compounds in a very short time. The first pass metabolism by the liver is lacking in *C. elegans*. Hence, to mimic the human active extract composition as closely as possible in the *C. elegans* test system, we chose to test both a cannabinoid-(and terpenoid) rich extract (i.e., non-polar fraction; NPF) as well as a cannabinoid-poor extract, enriched in polar compounds like flavonoids (i.e., polar fraction, PF). NPF extracts were made from dried flower tops extracted with *n*-hexane From the left-over flower material of this hexane extraction, the PF was made by extracting the dried left-overs with 70% EtOH. The cannabinoid concentration in the PFs was about 500–1000 times lower as compared to the NPF.

**Fig. 1:**
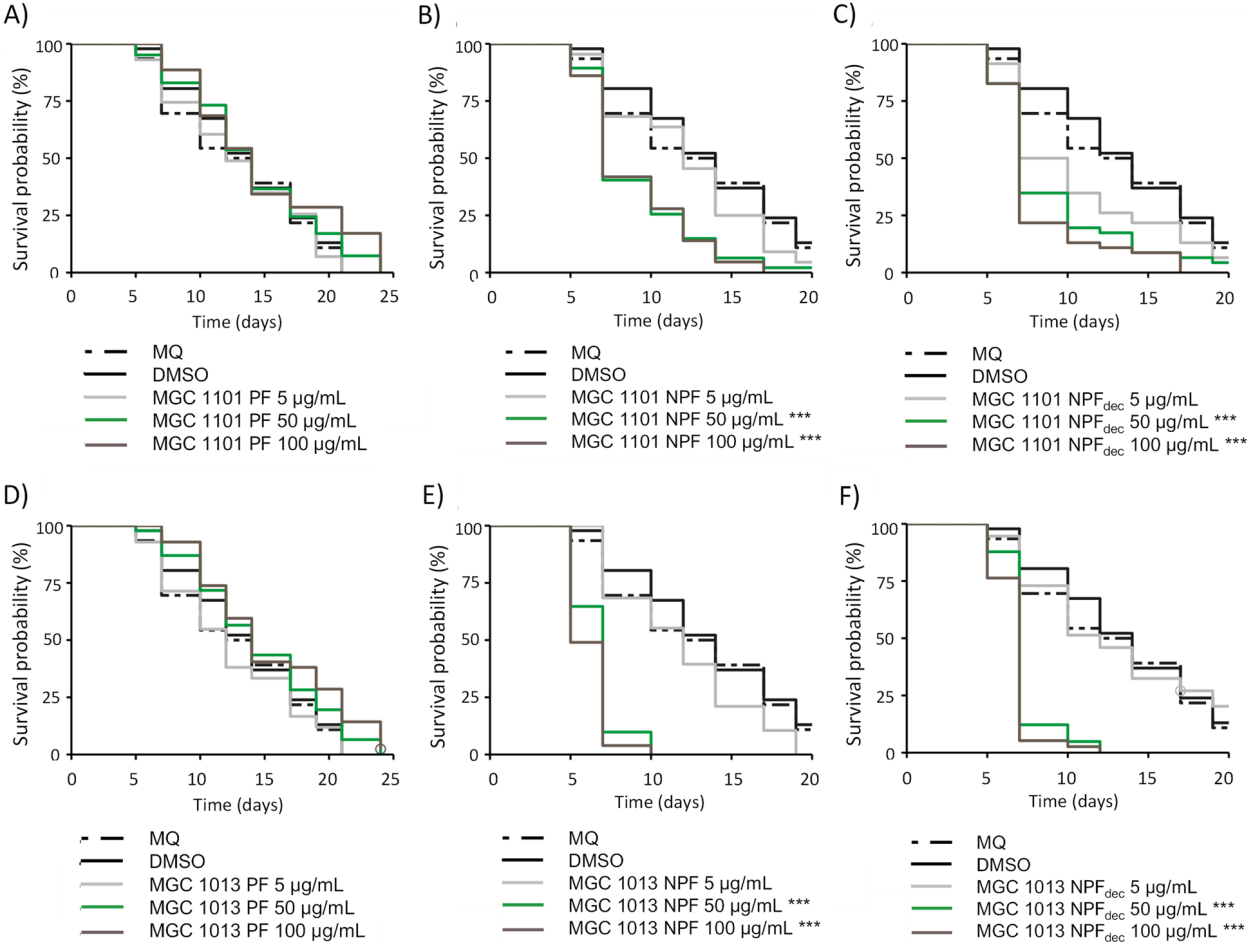
Kaplan-Meier survival rate of *C. elegans* exposed to different types of cannabis. *C. elegans* was exposed to 5, 50, and 100 μg/mL extracts obtained from the Δ^9^ -tetrahydrocannabinol (THC)-rich cannabis variety MGC 1101 (**A**–**C**) and the cannabidiol (CBD)-rich variety MGC 1013 (**D**–**F**). Vehicle control (Milli-Q water (MQ)/dimethyl sulfoxide (DMSO)) and different doses of **A** polar fraction (PF) of MGC 1101; **B** non-polar fraction (NPF) of MGC 1101; **C** decarboxylated non-polar fraction (NPF_dec_) of MGC 1101; **D** PF of MGC 1013; **E** NPF of MGC 1013; **F** NPF_dec_ of MGC 1013. Kaplan-Meier survival analysis was used to compare the vehicle control (DMSO) condition to the experimental conditions. *=false discovery rate (FDR)-corrected *p* < .05; *** = FDR-corrected *p* < .001

To investigate the optimal test concentrations of the different extracts with *C. elegans* as a test model, a life span assay was performed as an initial test. *C. elegans* nematodes were exposed to 5 μg/mL, 50 μg/mL, or 100 μg/mL of either NPF extracts in its carboxylated- (NPF; containing mainly acidic cannabinoids) and decarboxylated state (NPF_dec_: exposed to heating and mimicking the effect of smoking), or to the PF extracts. One THC-rich (MGC 1101 in Fig. 1A–C) and one CBD-rich (MGC 1013 in Fig. 1D–F) variety was tested. The total THC concentration in the extracts of MGC 1101 was 277 μg/mg extract for the NPF and 0.4 μg/mg for the PF. The CBD concentration in the extracts of MGC 1013 was 81 μg/mg for the NPF and 0.14 μg/mg in the PF. Survival probability was monitored during the life span of the nematodes as compared to the vehicle control (0.5% EtOH).

Toxic effects of the NPF were observed in nematodes at concentrations of 50 μg/mL and 100 μg/mL of THC-and CBD-rich extracts, independent of its decarboxylated state. Polar fractions did not induce toxic effects as compared to the control nematodes. As PFs did not induce toxicity over the complete range of concentrations, investigations towards variety-specific effects on different health endpoints were mainly performed using the cannabinoid-poor PF extracts.

### Chemical profiling of different varieties of cannabis

**Fig. 2:**
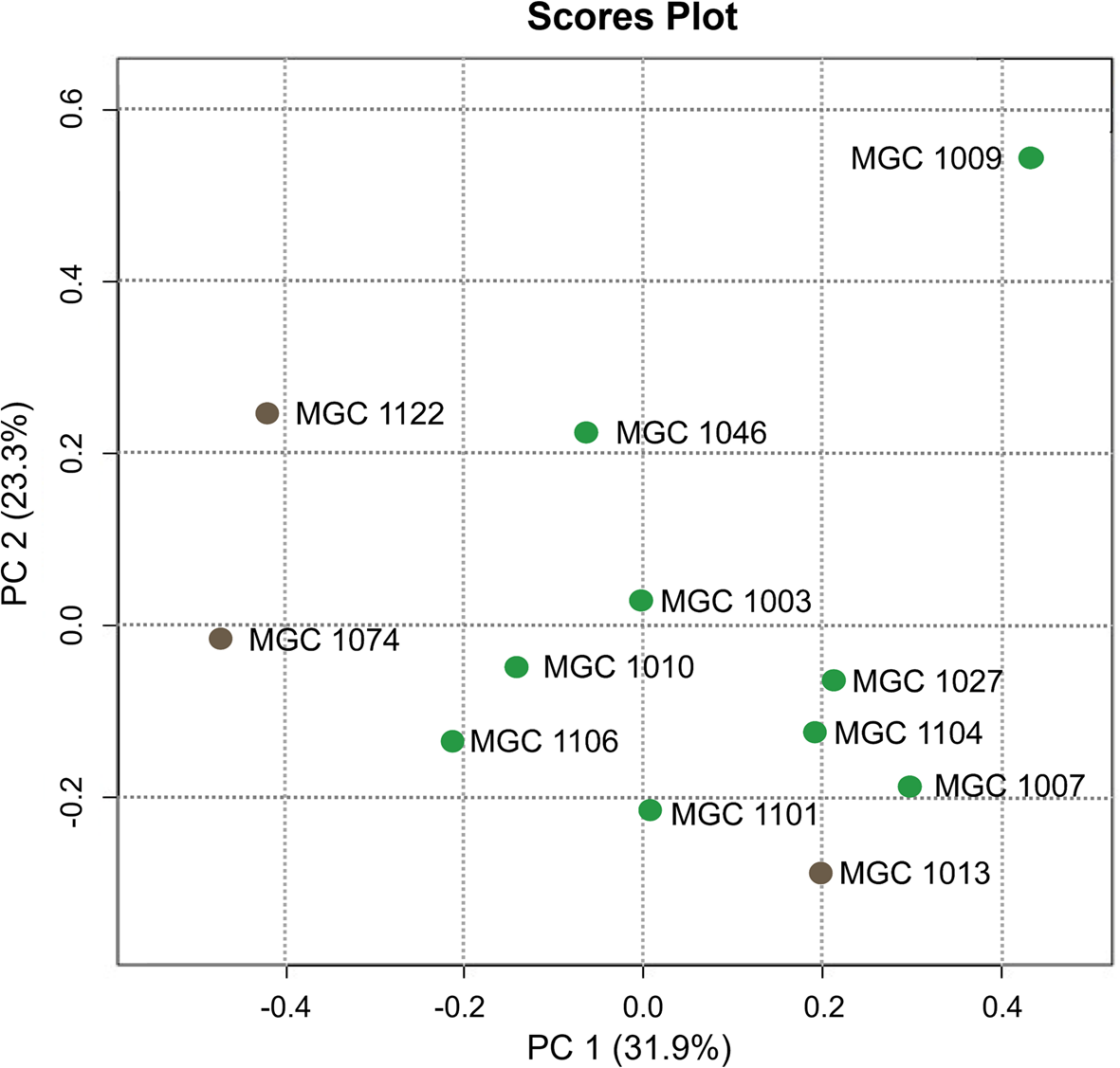
Principal component analysis (PCA) score plot of the 12 different genotypes based on ^1^H-NMR profiles of polar extracts (PF). This graph shows the differences in metabolic profiles of compounds in the extracts of the different varieties. PCA was performed using MetaboAnalyst 5.0 with auto-scaling. Green dots denote Δ^9^-tetrahydrocannabinol (THC)-rich varieties; brown dots denote cannabidiol (CBD)-rich varieties

The different health-beneficial effects of the individual varieties might be explained by differences in chemotype. To compare constituents from varieties, chemical profiles of the polar fractions (PFs) of the different varieties were generated with NMR (Additional file S2). Principal component analysis (PCA) of the NMR results indeed showed that the varieties could not be clustered and are quite different from each other with respect to their metabolic composition (Fig. 2).

### Bioactivity of cannabinoid-poor cannabis extracts in the model organism *C. elegans*

To investigate the broad health-application range of cannabis, research with *C. elegans* was clustered in three classes relevant to the human subjective experience with cannabis: appetite control, motility, and nervous system function (memory and the response to an acute adverse stimulus).

#### Appetite control: Cannabis influences *C. elegans* pharyngeal pumping in a variety-specific manner

Appetite in *C. elegans* can be investigated by measuring pharyngeal pumping: the neuromuscular contraction of the pharynx. *C. elegans* pharyngeal pumping occurs during food intake and is influenced by previous food experiences (for example, starvation and/or food quality) (Ishita et al. 2020). Pharyngeal contractions lead to grinding of the food and food intake (Avery and You 2012; Trojanowski et al. 2016).

**Fig. 3:**
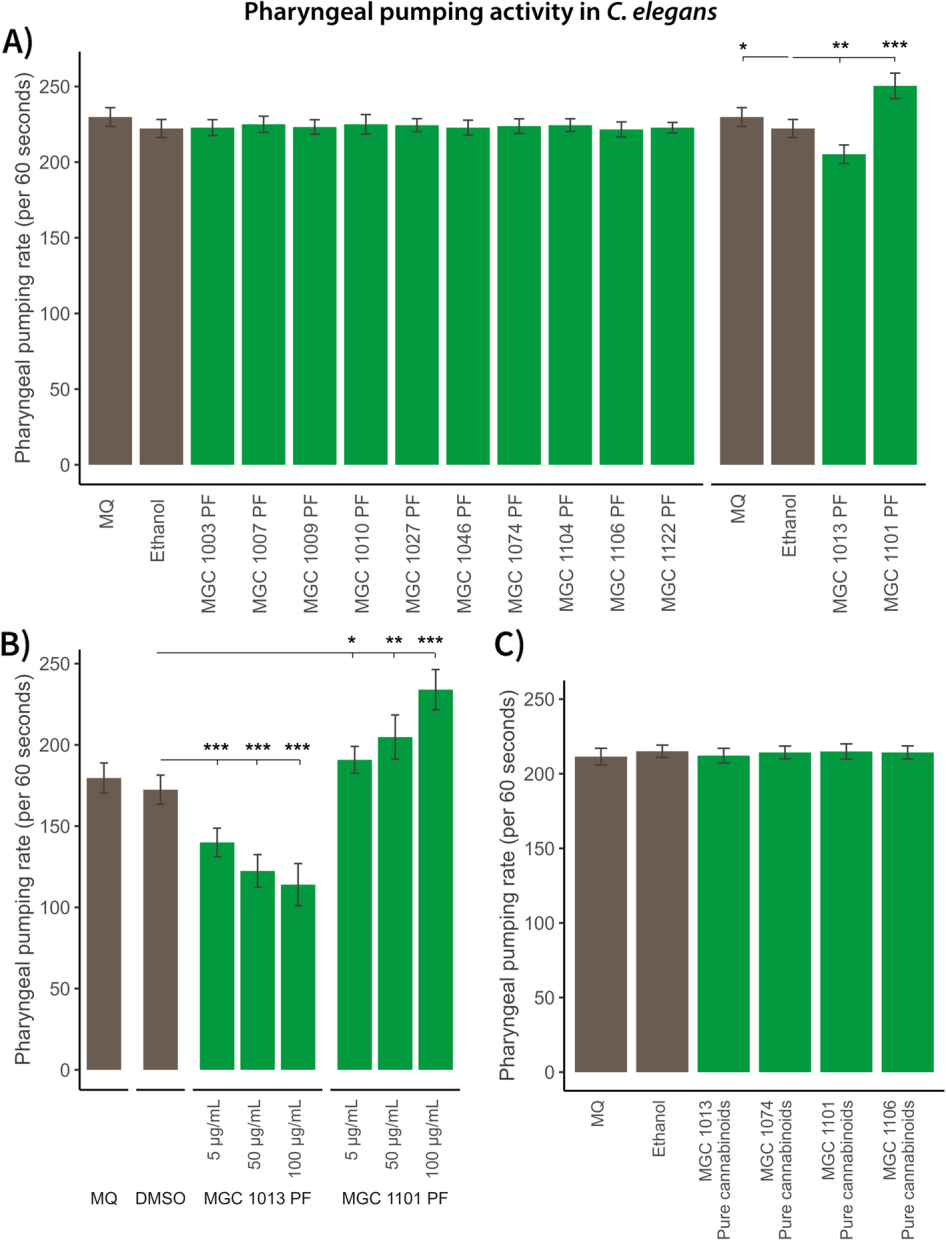
Effect of polar fractions (PFs) of different cannabis varieties on pharyngeal pumping activity in *C. elegans*. The nematode *C. elegans* was exposed to the PFs from different cannabis varieties as well as pure cannabinoids. **A** Overview of pharyngeal pumping activity of 100 μg/mL PF of all cannabis varieties used in this study; **B** dose-response with 5, 50, and 100 μg/mL PF extracts of the two varieties MGC 1101 and MGC 1013 that showed an effect on the pharyngeal pumping activity; **C**: pharyngeal pumping activity in nematodes treated with pure cannabinoids containing 0.0143 μg/mL cannabidiol (CBD) (similar to the concentration in the polar fraction (PF) of MGC 1101), pure cannabinoids containing 0.0125 μg/mL CBD (similar to the concentration in the PF of MGC 1074), pure cannabinoids contain 0.0262 ug/mL Δ^9^-tetrahydrocannabinol-acid (THCA) and 0.0133 μg/mL Δ^9^-tetrahydrocannabinol (THC) (similar to the concentration in the PF of MGC 1101), and pure cannabinoids contain 0.0278 μg/mL THCA and 0.0140 μg/mL THC (similar to the concentration in the PF of MGC 1106). Mann-Whitney *U* tests (**A**) and *t* tests (**B**) were used to compare the vehicle control conditions ethanol (**A**) and Dimethyl sulfoxide (DMSO) (**B**) to the experimental conditions. Milli-Q water (MQ) is the water control in A and B. Green bars denote pharyngeal pumping activity in *C. elegans* exposed to polar fractions (PFs) of cannabis; brown bars denote vehicles; *** = false discovery rate (FDR)-corrected *p* < .001; ** = FDR-corrected *p* < .01; * = FDR-corrected *p* < .05

Nematodes were grown from L1 to young adult stage on solid agar plates that contained 100 μg/mL of the PF of the different cannabis varieties. Pharyngeal pumping was tested by measuring the number of pharyngeal pumps per minute. We found that pharyngeal pumping was only altered after treatment with two of the 12 varieties, of which one derived from the CBD-rich variety MGC 1013 and one derived from the THC-rich variety MGC 1101 (Fig. 3A). In both cases, the deviated pharyngeal pumping activity was dose-responsive (Fig. 3B). This suggests that only two of the studied varieties could be used in indication areas where eating behavior and appetite play an important role: MGC 1013 for appetite reduction and MGC 1101 for appetite induction. Interestingly, the PFs of MGC 1074 with a CBD content equal to MGC 1013 and MGC 1106 with a THC/THCA content equal to MGC 1101 (see Additional file S6) did not affect the pharyngeal pumping activity indicating that the cannabinoids might not be involved in this activity. This was confirmed by exposing *C. elegans* to mixtures of pure cannabinoids (see Additional file S7 for purity check) representing the cannabinoid concentrations in MGC 1013, MGC 1074, MGC 1101, and MGC 1106. No effect on the pharyngeal pumping activity was detected (Fig. 3C). Pharyngeal pumping was reduced in both MGC 1013 and MGC 1101 after nematode treatment with a seemingly non-toxic dose of 5 μg/mL NPFs (Additional file S3). NPFs do not only contain cannabinoids but also terpenoids and other non-polar compounds so we cannot completely exclude specific NPF effects. However, life span curves after nematode treatment with this concentration of NPF showed a mild reduced survival probability (Fig. 1). Furthermore, increased concentrations of NPF further reduced pharyngeal pumping in both varieties (Additional file S3) so reduction in pharyngeal pumping effects in NPF-treated nematodes might also be reflective for a mild initial toxicity response.

#### Effects of cannabis on motility and body oscillation

**Fig. 4:**
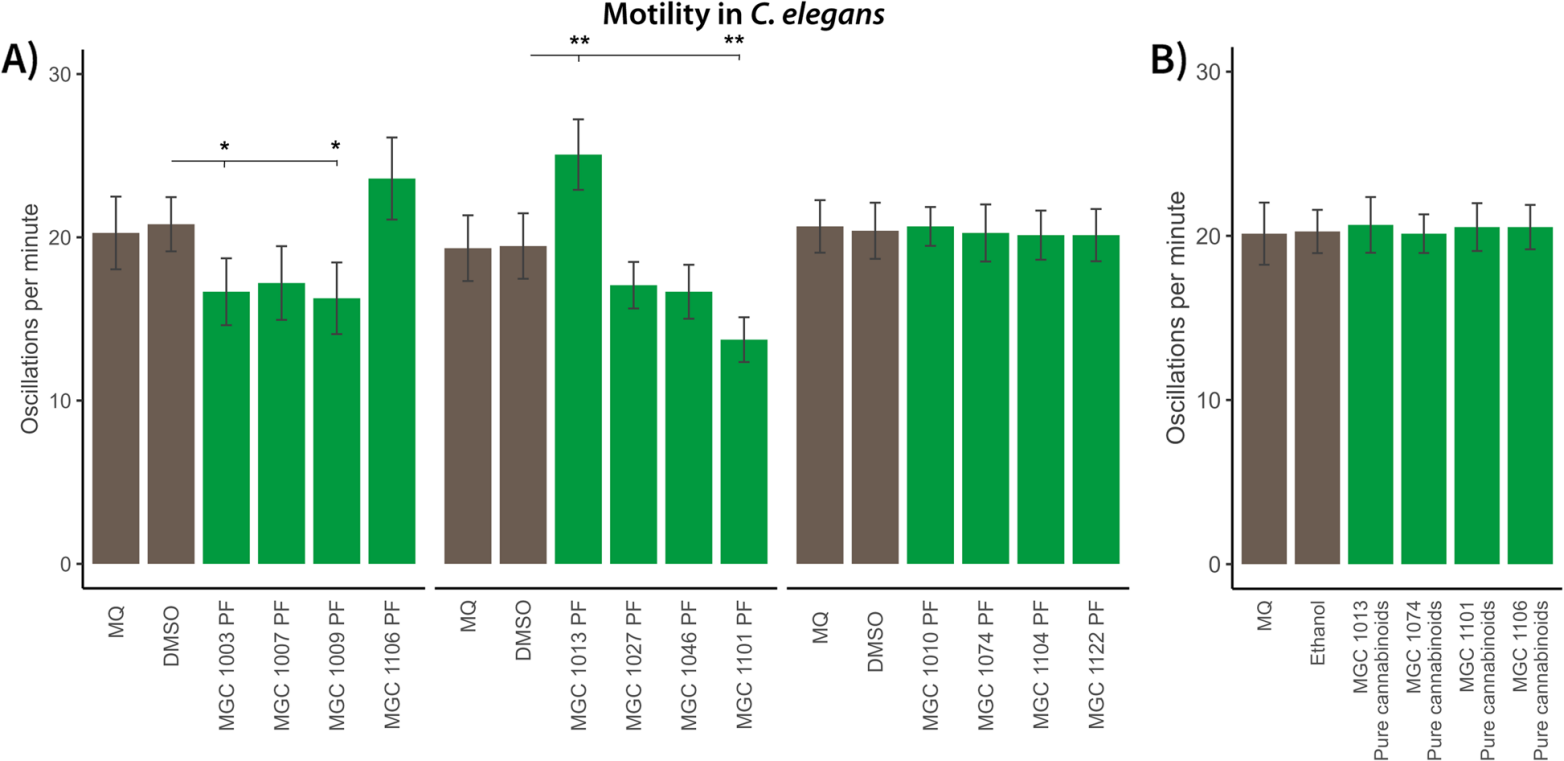
Effect of polar fractions (PFs) from different varieties on *C. elegans* motility. The number of full body bends (oscillations) per minute is used as measure for motility after treatment with different varieties from L1 to young adult stage. **A** effect on motility after exposure of *C. elegans* to 100 μg/mL PFs of all varieties used in this study: MGC 1003, MGC 1009, and MGC 1101 induce reduced motility in *C. elegans* after treatment, MGC 1013 increased motility; **B** effect on motility after exposure of *C. elegans* to pure cannabinoids containing 0.0143 μg/mL cannabidiol (CBD) (similar to the concentration in the polar fraction (PF) of MGC 1101), pure cannabinoids containing 0.0125 μg/mL CBD (similar to the concentration in the PF of MGC 1074), pure cannabinoids contain 0.0262 μg/mL Δ^9^-tetrahydrocannabinol acid (THCA) and 0.0133 μg/mL Δ^9^-tetrahydrocannabinol (THC) (similar to the concentration in the PF of MGC 1101), and pure cannabinoids contain 0.0278 μg/mL THCA & 0.0140 μg/mL THC (similar to the concentration in the PF of MGC 1106). Mann-Whitney *U* tests were used to compare the vehicle control conditions Dimethyl sulfoxide (DMSO) to the experimental conditions. Milli-Q (MQ) is the water control. Green bars denote motility of *C. elegans* exposed to PFs of different cannabis varieties; brown bars denote vehicles; ** = false discovery rate (FDR)-corrected *p* < .01; * = FDR-corrected *p* < .05

Cannabis is known to exert a relaxing effect on the body. Therapeutic cannabis is used for muscle relaxation in relation to spasms (Mack 2000). To test whether the different genotypes influence muscle function, a motility was performed. *C. elegans* nematodes were treated with 100 μg/mL PF of the different varieties from L1 to young adult stage. Muscle function was assessed by scoring the number of oscillations (full body bends) of nematodes per minute (see Fig. 4A). Variety-specific differences on motility were observed. While the motility of nematodes treated with PFs from the varieties MGC 1003, MGC 1009, and MGC 1101 was significantly reduced, MGC 1013-treated nematodes showed enhanced movement patterns. Like in the previous pharyngeal pumping assay, enhanced motility of nematodes after treatment with the PF of MGC 1013 could not be explained solely due to CBD levels as nematode treatment with extracts derived from other CBD-rich varieties MGC 1074 and MGC 1122 did not influence muscle activity. Moreover, treatment with pure CBD in the same concentration as present in MGC 1013 did not induce an effect (Fig. 4B). Oppositely, reduced motility could not explained solely by THC levels. While nematode treatment with PF derived from most THC-rich varieties showed reduced physical activity, not all PF of THC-rich varieties showed this effect. Treatment with a combination of THCA and THC in the same concentration as present in MGC 1101 did not show any effect on motility (Fig. 4B). Exposure to a low and non-toxic dose of cannabinoid-rich NPFs from the different varieties did not show any significant effect on the motility, as compared to the control, indicating that compounds present in the PF play an important role in motility in *C. elegans* (Additional file S4).

#### Effects of cannabis on the nervous system

The nervous system is responsible for the integration of external stimuli in order to induce appropriate behavioural and cognitive responses. Cannabis is known for its effects on the nervous system. Like other living organisms, *C. elegans* has to integrate external stimuli in order to respond in an appropriate manner to its surroundings. Cognitive relaxation can therefore be defined by the reduced response towards environmental signals that depend on their integration through the nervous system. When *C. elegans* is exposed to noxious odorants like 1-octanol, nematodes respond with acute avoidance behaviour and they move backwards when the odor is placed in front of the head (Chao et al. 2004).

**Fig. 5:**
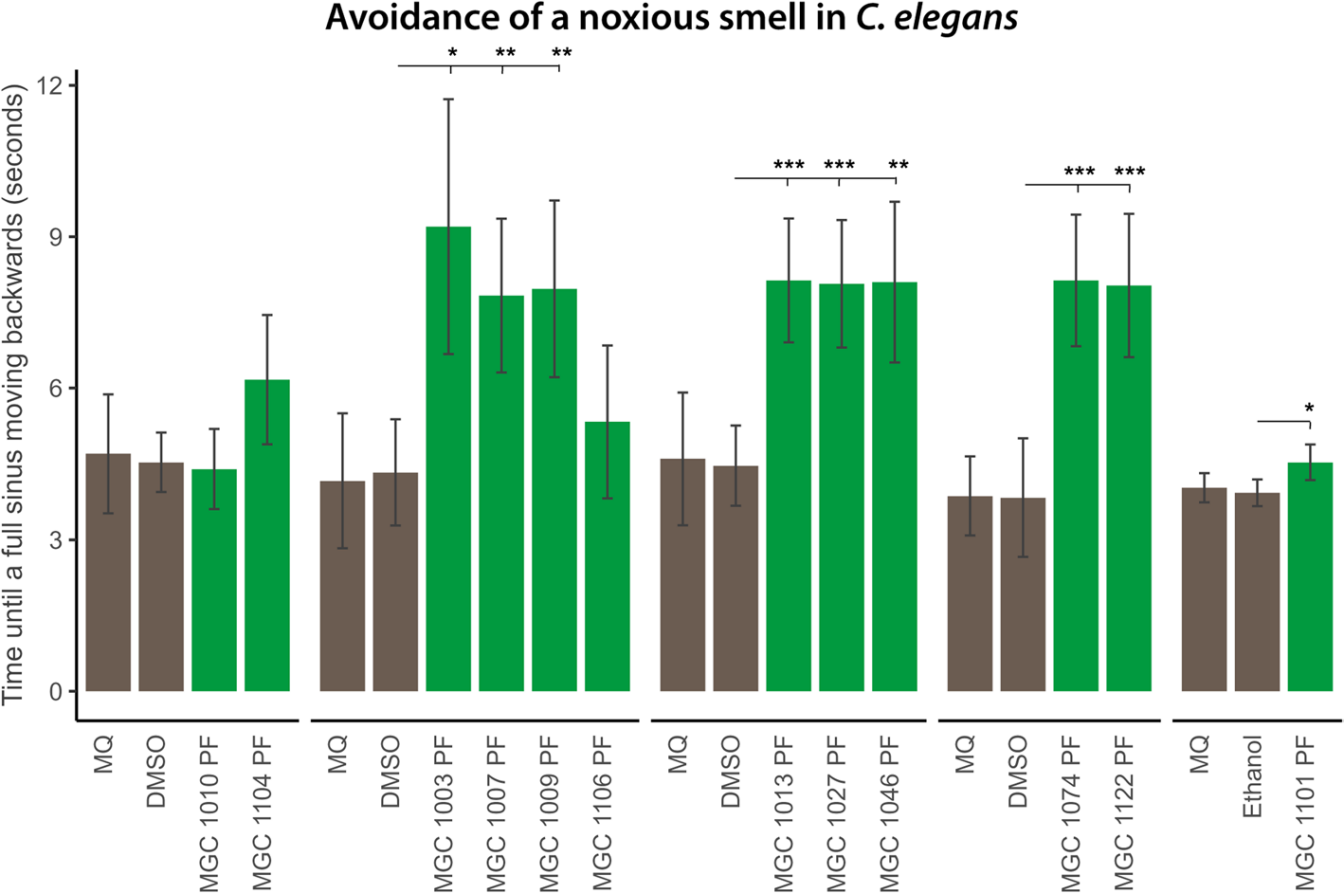
Effect of polar fractions (PFs) of different varieties on the avoidance of a noxious smell. The effect of the polar fractions (PFs) on the nervous system was assessed by the effect on the time of one full reversal backwards movement of 1-octanol treated *C. elegans* exposed to 100 μg/mL PFs of different varieties. Treatment with PF of varieties: MGC 1003, MGC 1007, MGC 1009, MGC 1013, MGC 1027, MGC 1046, MGC 1074, MGC 1122, and MGC 1101 all reduced the responsiveness of nematodes to the aversive odor 1-octanol. MQ indicates the Milli-Q water control group, DMSO (dimethyl sulfoxide) and ethanol the vehicle control. Mann-Whitney *U* tests were used to compare the vehicle control conditions (DMSO/ethanol) to the experimental conditions. Green bars denote the effect on avoidance by *C. elegans* exposed to PFs of different cannabis varieties; brown bars denote vehicles; *** = false discovery rate (FDR)-corrected *p* < .001; ** = FDR-corrected *p* < .01; * = FDR-corrected *p* < .05

To investigate effects of different varieties on effects on the nervous system, PFs from different varieties were tested for the reverse movement response to 1-octanol. In this assay, L1 nematodes were grown to young adulthood in either the presence or absence of PF. During the assay, an eyelash dipped in 1% 1-octanol was held at one body distance in front of individual nematodes. The response to 1-octanol was measured as the time that it took them to complete one full sinusoidal body reversal. The experiment was performed in duplicate with 15 nematodes in each condition. The results are shown in Fig. 5. Exposing the nematodes to the PFs of the varieties MGC 1003, MGC 1007, MGC 1009, MGC 1013, MGC 1027, MGC 1046, MGC 1074, MGC 1122, and MGC 1101 reduced the response to 1-octanol statistically significantly, as compared to the control. Thus, polar fractions of most varieties influenced the nematode responsiveness towards an unpleasant environmental stimulus. Interestingly, exposing the nematodes to a low and non-toxic dose of NPF and NPF_dec_ showed only effects in two CBD-cultivars: MGC 1074 and 1122 (Additional file S5).

Due to the fact that the aversive response of nematodes to 1-octanol is scored by assessing motility, the delayed backward response of nematodes treated with PFs of MGC 1003, MGC 1009, and MGC 1101 might simply represent a secondary effect of movement deficits due to treatment, e.g., “stonedness”. This reduced response to 1-octanol however is clearly less strong when nematodes were treated with MGC 1101. Even though this variety reduced overall motility, e.g., increased muscle relaxation (Fig. 4), nematodes maintain the ability to respond more easily to unpleasant environmental stimuli by moving away from it when compared to other affected varieties. MGC 1101 might therefore be beneficial for a very wide group of patients who benefit from muscle relaxation. All PFs obtained from the CBD-rich varieties MGC 1013, MGC 1074, and MGC 1122 showed a reduced sensitivity to adverse 1-octanol. However, nematode treatment with NPFs of MGC 1013 did not show this effect while in MGC 1074 and MGC 1122 the reduced sensitivity to 1-octanol was still present. This suggests that the NPF of MGC 1013 lacks the compounds that influence the sensitivity to 1-octanol. The fact that PF and NPF of CBD varieties can influence nervous system signalling is in line with the fact that CBD is known for its mind-relaxing properties in humans (Melas et al. 2021; Shannon et al. 2019).

**Fig. 6:**
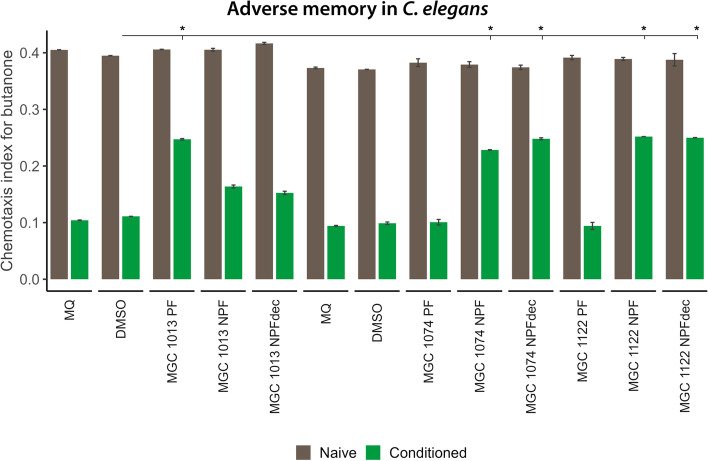
Effect of cannabis fractions on adverse memory. The effect of polar fractions (PFs; 100 μg/mL) and a non-toxic concentration of the non-polar fractions (decarboxylated (NPF_dec_; 5 μg/mL) and non-decarboxylated (NPF; 5 μg/mL) of the cannabidiol (CBD)-rich cannabis variety MGC 1013, MGC 1074 and MGC 1122 was tested on adverse memory with conditioning in the presence or absence of butanone. Brown bars indicate the naïve population, green bars the adversely butanone conditioned treatment groups. The PF of MGC 1013 as well as the NPF of MGC 1074 and MGC 1122 reduce the avoidance behavior of nematodes towards butanone (as can be seen by a higher chemotaxis level in conditioned group). MQ indicates the Milli-Q water control group, DMSO (dimethyl sufoxide) the vehicle control. *t* tests were used to compare the vehicle control conditions (DMSO) to the experimental conditions for each learning condition. * = false discovery rate (FDR)-corrected *p* < .05

To further investigate the effects of CBD-rich varieties on nervous-system-functioning with regard to adverse stimuli, nematode responses to an adverse memory stimulus were assessed using an adverse memory assay. If *C. elegans* nematodes are placed in an adverse condition (for example the absence of food) and in the presence of butanone, nematodes develop negative memory towards this butanone smell (i.e., negative conditioning). When re-encountering the smell, nematodes respond by avoiding the smell and migrating away from it. The aversive encounter also induces stress responses in the body (Eliezer et al. 2019). The response of nematodes towards adverse conditioning was tested by treatment of nematodes with the PF and NPF derived from CBD-rich varieties MGC 1013, MGC 1074, and MGC 1122 (see Fig. 6).

While MGC 1013 PF extracts indeed show a reduced stress response with regards to adverse memory triggers, the PF extracts of MGC 1074 and MGC 1122 do not have the ability to reduce the memory-related stress response. NPF extracts of MGC 1074 and MGC 1122, however, did reduce stress responses towards the adverse memory trigger. We concluded that, even though all extracts derived from CBD-rich varieties seem to play a role in influencing the nervous system response towards adverse environmental signals, they do this in different manners. PF of MGC 1074 and MGC 1122 can influence nervous system signalling in *C. elegans* with regards to adverse smell but do not influence adverse memory responses. NPF of these cultivars have a broader effect and influence both the response to adverse smell and adverse memory. For MGC 1013 however, only the PF influences both the response to adverse smell and adverse memory. It will be of interest to further disentangle the effects of CBD varieties on nervous system signalling to enable targeted treatment of, for example, post-traumatic stress disorder.

**Fig. 7:**
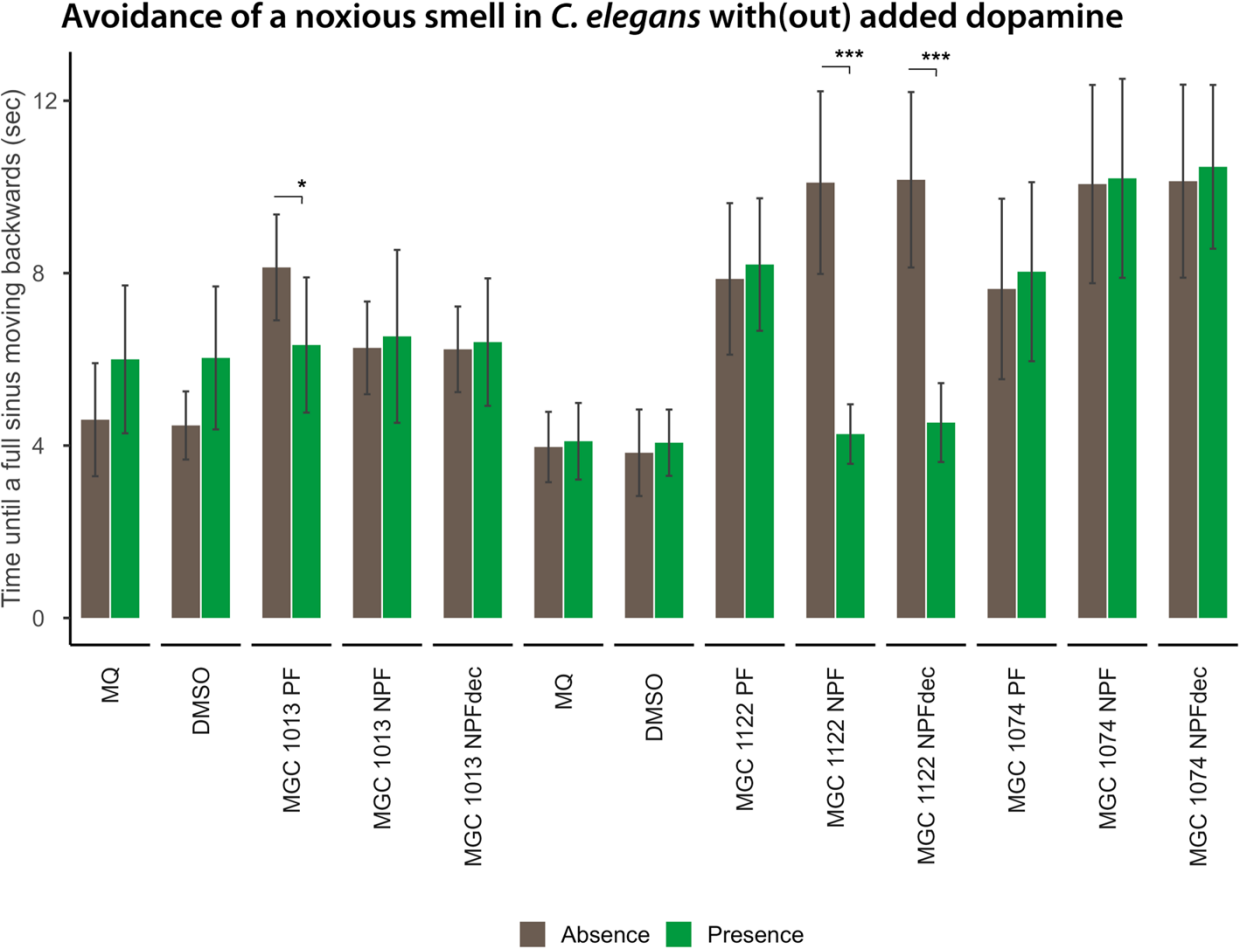
Influence of cannabis fractions on dopamine as mechanism to regulate the avoidance behavior towards 1-octanol. Effects of nematode treatment with the 100 μg/mL of the polar fractions (PFs) of the cannabidiol (CBD)-rich cannabis varieties MGC 1013, MGC 1074, and MGC 1122 on the avoidance of 1-octanol in the presence (green bars) or absence (brown bars) of added dopamine. Nematode treatment with the 100 μg/mL PF of MGC 1013 as well as treatment with a non-toxic dose of 5 μg/mL of the non-polar fraction (NPF) of MGC 1122 alleviates the delayed response to 1-octanol induced by the treatment, suggesting that treatment leads to reduced levels of dopamine and thereby to delayed responsiveness towards aversive 1-octanol. MQ indicates the Milli-Q water control group, DMSO (dimethyl sulfoxide) the vehicle control. Mann-Whitney *U* tests were used to compare each condition without added dopamine to the same condition with added dopamine. *** = False Discovery Rate (FDR)-corrected *p* < .001; * = FDR-corrected *p* < .05

It is known that the response to 1-octanol is under control of dopamine and glutamate signalling. The sensitivity to 1-octanol is reduced when dopamine levels are low or when glutamate signalling is enhanced (Chao et al. 2004; Baidya et al. 2014). To assess whether a lack of dopamine could be the cause of the delayed sensitivity of MGC 1013-, MGC 1074-, and MGC 1122-treated nematodes to 1-octanol, the 1-octanol test was repeated in the presence of externally added dopamine in polar and non-polar fractions (50 mM; Baidya et al. 2014) (see Fig. 7).

The reduced sensitivity to 1-octanol was restored in MGC 1013 PF- and MGC 1122 NPF-treated nematodes after external addition of dopamine. This indicates that the delayed response to a noxious smell in nematodes treated with these extracts is likely caused by reduced dopamine levels in treated nematodes. The reduced sensitivity to 1-octanol was not restored after MGC 1074 (PF and NPF) treatment in the presence of dopamine and thus is likely regulated by other molecular signalling routes.

Overall, MGC 1013 seems to be a scientifically interesting variety in appetite, body, and nervous system function. The pattern of health endpoints influenced by MGC 1013 were similar to health benefits that are also induced by dietary restriction. Dietary restriction leads to prolonged life span in many organisms and increases in overall fitness (Green et al. 2022; Kapahi et al. 2017). MGC 1013 treatment in *C. elegans* caused reduced feeding as well as a slight not-significant prolonged life span. Treatment with MGC 1013 also increased motility of nematodes, which indicates enhanced overall fitness.

**Fig. 8:**
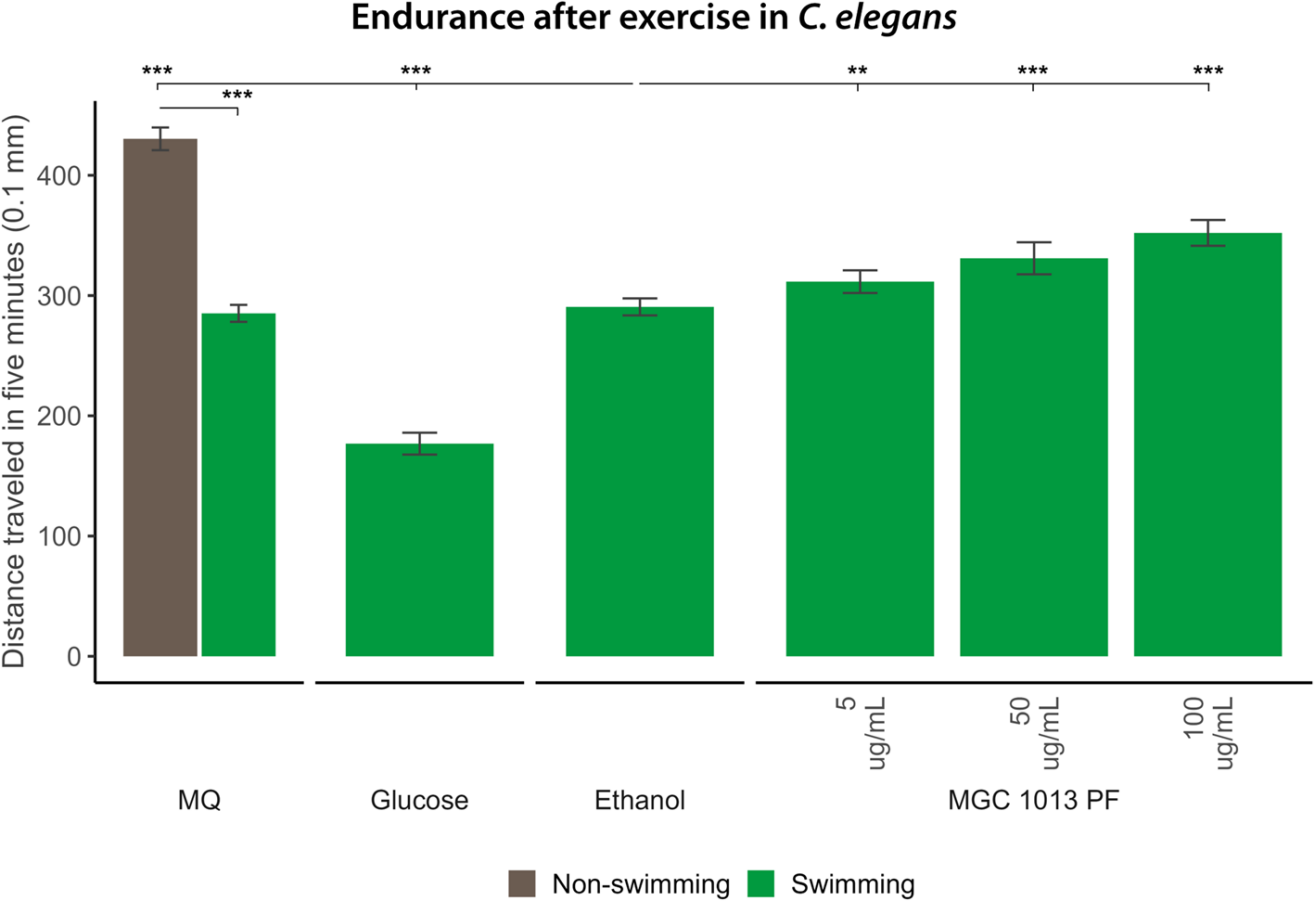
Effect of nematode treatment with the polar fraction (PF) of MGC 1013 on endurance after exercise. Nematodes treated with different concentrations of 5, 50, and 100 μg/mL of the PF of MGC 1013 were forced to swim during 120 min. After this the distance travelled within the first 5 min on a solid agar medium was scored. After swimming nematodes travel a shorter distance (green Milli-Q water (MQ) untreated control group) when compared to the non-swimming control group (brown bar). Glucose treatment does not enhance performance. Nematode treatment with the PF of MGC 1013 enhances the travel distance of nematodes after swimming in a dose responsive manner, indicative for enhancing overall fitness or energy levels. Mann-Whitney *U* tests were used to compare the vehicle control conditions (ethanol) to all other conditions, as well as to compare MQ conditions between exercise and rest. Brown bars denote the rest condition (non-swimming); green bars denote the exercise condition (swimming); *** = false discovery rate (FDR)-corrected *p* < .001

To investigate whether MGC 1013 indeed enhances fitness, energy levels of MGC 1013-treated nematodes were compared to untreated ones. For this analysis, *C. elegans* nematodes were grown in the presence of PF of MGC 1013 from L1 until young adult stage. When mature, nematodes were challenged to swim for 2 h, after which their remaining energy was monitored by measurement of the travelled distance covered in the first 5 min after the exercise. Results are shown in Fig. 8 and confirm that treatment with the PF of MGC 1013 indeed seems to enhance overall energy levels and fitness. Thus, treatment with MGC 1013 might be relevant to improve fitness and health.

## Discussion

Cannabis is still a prohibited substance worldwide and the distribution of medicinal cannabis to patients (e.g., by doctor’s prescription) is strictly regulated in most countries. As far as we know, the breeding programs and quality control of legal medicinal cannabis is mainly focused of the major cannabinoids and not on other bioactive compounds present in the cannabis matrix. The fact that cannabis is beneficial for a very broad spectrum of diseases, and that patients prefer a specific cannabis variety for treating their specific disease, already implies that not just the major cannabinoids—like THC and/or CBD—are responsible for the beneficial effects. This phenomenon can be explained by the entourage effect of cannabis. In general, the data obtained from patients’ experiences are rather subjective and the existence of an entourage effect of cannabis is still under debate in the scientific literature (Cogan 2020; Russo 2018). The use of objective animal test models might contribute to a better understanding of the putative cannabis entourage effect.

Potential test models to study entourage effects of cannabis extracts like zebrafish embryo’s and *C. elegans* are already used as an essential tool in the pharmaceutical research industry to identify novel drugs and drug targets, and to study the molecular mechanisms behind the mode of action of drugs or complex herbal mixtures. The endocannabinoid system including the CB1 and CB2 receptors are present in zebrafish; and recently, the zebrafish model system was successfully applied to study the health-promoting effects of cannabinoids as compared to cannabis extracts (Nixon et al. 2021; Bailone et al. 2022). The use of nematode *C. elegans* for studies to the health effect and mode of action of complex plant extracts has also some advantages: nematodes are genetically identical, can be studies throughout their complete life cycle, are small, low cost, and have a well-documented and thoroughly mapped nervous system with comparable neurotransmitters like in the human brain. The *C. elegans* research community has been well established and possibilities for high-throughput screening, and use of mutants and reporter strains are readily available. However, orthologs for CB1 and CB2 are missing in *C. elegans*. The protein NPR-19 in *C. elegans* is involved in endocannabinoid signalling and shares some homology with the CB1 receptor (Oakes et al. 2017). Key amino acids that are very likely involved in the interaction between CB1 and THC are however not conserved between NPR-19 and CB1 (Aviz-Amador et al. 2021) even though some of the residues involved in docking of the THC molecule in the CB1 receptor are conserved between the *C. elegans* NPR-19 and the human CB1 receptor. So currently, no clear evidence is available on whether THC can act through CB1 and CB2 like receptors in *C. elegans*. For CBD, however, it became recently more clear that other receptors than CB1 and CB2 are involved in (endo)cannabinoid signalling in humans. The vanilloid-type TRP channels as well as the serotonin receptor 5-HT_1A_ are known to be associated with a broad scale of effects, including neuroprotective effects of CBD (Pazos et al. 2013; de Almeida and Devi 2020). Orthologs of both TRP and 5-HT_1A_ are present in *C. elegans (*Xiao and Xu 2011*;* Pereira-Sousa et al. 2021*)*. Especially SER-4, the ortholog for the serotonin receptor 5-HT_1A_ is very interesting in this respect; several recent studies have shown that CBD has an effect on preventing neurodegenerative diseases and on longevity in *C. elegans* (Wang et al. 2021; Zhang et al. 2022; Frandsen and Narayanasamy 2022; Land et al. 2021).

When studying entourage effects the high cannabinoid concentrations in cannabis compared to the concentrations of other potential bioactive compounds might be an issue. In addition, to our knowledge, there is no or only very limited information available on the pharmacokinetics of full cannabis extracts, and the interaction of cannabinoids with other compounds and most of the current pharmacokinetic studies on cannabis are focused on cannabinoids only. From pharmacokinetic studies in humans, it is clear that cannabinoids are metabolized extensively by P450 enzymes at the first pass metabolism by the liver after oral administration. Smoking of cannabis results in a high peak of cannabinoids in the blood immediately after administration, but this peak drops dramatically within an hour (Lucas et al. 2018; Huestis et al. 1992). This implies that the ratio of cannabinoids and the other bioactive molecules present in cannabis may change dramatically shortly after administration and as a consequence these non-cannabinoid bioactive molecules might contribute significantly to the reported health effects.

In this report, we assessed whether the nematode *C. elegans* could be used as model for studying putative entourage effects of cannabis. The starting point was to select a broad set of varieties that were indicated by cannabis users to be beneficial for different health-promoting effects (see Additional file S10). In our study, we tested cannabinoid/terpenoid-poor extracts with enhanced levels of polar compounds on the one hand, and cannabinoid/terpenoid-rich extracts on the other hand. This enabled us to study both the cannabinoids in a low and non-toxic dose as well as the polar compounds in a high dose which might possibly reflect the situation in the body shortly after oral administration of cannabis. The effects of these extracts were tested in a set of bioassays. To our surprise, a broad set of biological effects related to appetite control, motility, body oscillation, and nervous system function was observed by exposing *C. elegans* to the polar fractions (PFs) of the different selected varieties. These effects are summarized in Table 1.

**Table 1:** Summary of the biological effects of polar fractions (PFs) of cannabis in *C. elegans*

Variety	***Appetite control***	***Motility***	***Nervous system*** ***(adverse acute stimulus)***	***Nervous system*** ***(adverse memory)***
MGC 1003	–	↓	↓	n.d.
MGC 1007	–	–	↓	n.d.
MGC 1009	–	↓	↓	n.d.
MGC 1010	–	–	–	n.d.
MGC 1013	↓	↑	↓	↓
MGC 1027	–	–	↓	n.d.
MGC 1046	–	–	↓	n.d.
MGC 1074	–	–	↓	–
MGC 1101	↑	↓	↓	n.d.
MGC 1104	–	–	–	n.d.
MGC 1106	–	–	–	n.d.
MGC 1122	–	–	↓	–

PFs of different cannabis varieties were used to test the effect on appetite control, body oscillation, motility, and nervous system function in *C. elegans.* (↓) significant decreased effect; (↑) significant increased effect; (–): no significant effect; *n.d.* not determined

This was an unexpected observation, since not only the concentration of cannabinoids was strongly reduced in the PFs, but also the concentration of terpenoids. Based on the results obtained by the PFs of the different varieties in the different bioassays, there are several indications that entourage effects indeed might play a role. First, the biological effects could not been explained by the remaining cannabinoids in the PFs since different varieties with a similar cannabinoid profile showed different effects in the bioassays. For example, the PFs of the varieties MGC 1013 and MGC 1101 have an effect on pharyngeal pumping activity (appetite control), whereas the PFs of the varieties MGC 1074 and MGC 1006—that have a similar profile of the major cannabinoids as in MGC 1013 and MGC 1101, respectively—did not show any effect on pharyngeal pumping activity. Second, when studying the muscle function, the PFs of only THC varieties MGC 1003, MGC 1009, and MGC 1101 showed reduced muscle activity (Fig. 4), and in addition the PF’s of most THC varieties except MGC 1004, MGC 1106, and MGC 1010 reduced nematode sensitivity towards an adverse outside stimulus (Fig. 5). Third, for the CBD varieties we tested the sensitivity towards adverse memory and identified that the individual CBD varieties regulate nervous system signalling through different means. While MGC 1013 and MGC 1122 reduce the overall levels/signalling of dopamine, MGC 1074 acts by different means. The occurrence of entourage effects was further supported by the fact that treating *C. elegans* with pure cannabinoid compounds in the same concentration and ratio as present in PFs of the bioactive varieties did not show any significant effect at all (Fig. 3 C and Fig. 4 B and Additional files S8, S9). Bioactivity was not only observed in the PF’s of the cannabis varieties. Certain nervous-system-specific effects of the CBD-rich varieties MGC 1074 and MGC 1122 do require non-polar terpenoid and cannabinoid-rich fractions (Additional file S5). Interestingly this activity as independent of the carboxylated status of the cannabinoids.

Our approach using separated PFs and NPFs in different *C. elegans* bioassays enhances the knowledge on the contribution of all relevant bioactive molecules to the health-promoting effect of cannabis and to study entourage effects. This is an essential factor in order to supply tailor-made medicinal cannabis for patients suffering from specific diseases. Although the nature of the data obtained from users of the selected medicinal varieties tested in this report are strictly anecdotal yet, the reported health effects are surprisingly similar with the effects we obtained with *C. elegans* especially exposed to the PFs of these varieties (see Additional file S10). Still, a more scientifically based or survey or controlled trial is necessary to confirm this overlap.

## Conclusion

In order to produce and supply medicinal cannabis that is specifically optimized for a broad spectrum of health-related applications, more knowledge and understanding is required about the contribution of all bioactive compounds present in cannabis extracts and their putative synergistic effects. Test model systems like *C. elegans*, in combination with metabolomics, seem versatile tools to identify all bioactive compounds in different cannabis varieties and to reveal the next steps in how entourage effects influence different health endpoints. This can be of high interest for developing and optimizing cannabis breeding programs, as well as for product development for different medicinal applications.

## Supplementary Information

**Additional file 1: Additional file S1.** Schematic overview of the agar assay plate used for the adverse memory assay. After treatment with cannabis extracts from L1 stage to young adulthood, nematodes were starved in the presence or absence of butanol (see material and methods). After a starvation period, nematodes were allowed to recover for several hours and then assessed for avoidance behavior towards butanol. Nematodes were placed in the middle of the plate and the amount of nematodes at B and E was scored after one hour. Quadrants E were immersed with sodium azide combined with 1% ethanol and quadrants B were immersed with sodium azide in combination with 1% butanone. Nematodes were pipetted in the center of the plate and the movements to the different quadrants after treatment with cannabis extracts was monitored.

**Additional file 2: Additional file S2.**^1^H-NMR spectra of the 12 different genotypes. (A) Full range of ^1^H-NMR spectra. Number indicates the different genotypes. The region (10.0-5.0 ppm) was expanded (B). 1: MGC 1046, 2: MGC 1010, 3: MGC 1007, 4: MGC 1074, 5: MGC 1027, 6: MGC 1122, 7: MGC 1004, 8: MGC 1009, 9: MGC 1003, 10: MGC 1006, 11: MGC 1001, 12: MGC 1013. The region where the metabolites typically appeared are mentioned in the figure such as amino acids, sugars and cannabinoids. *Phenolics indicates compounds containing a phenolic ring in the molecules.

**Additional file 3: Additional file S3.** Pharyngeal pumping activity of the Non-Polar Fractions (NPF) of the cultivars MGC 1013 and MGC 1101. In this assay *C. elegans* was exposed to different concentrations (5, 50 and 100 μg/mL) of the Non-Polar Fractions both in carboxylated (NPF_dec_) and non-decarboxylated (NPF) form. Nematodes were treated from L1 stage to young adulthood with NPF. Pharyngeal pumping frequency is plotted on the Y axis. Pharyngeal pumping activity of all fractions was decreased possibly due to toxicity (see Figure 1). The cannabinoid concentration in the 5 μg/ml NPF of MGC 1013 and MGC 1101 matches with the cannabinoid concentration in the 100 μg/ml PF fractions of MGC 1013 and MGC 1101 respectively (Figure 3). A one-way ANOVA and post-hoc Welch tests were used to compare the vehicle control condition Dimethyl sulfoxide (DMSO) to the experimental conditions. *** = False Discovery Rate (FDR)-corrected *p* < .001; ** = FDR-corrected *p* < .01.

**Additional file 4: Additional file S4.** Effect of Non Polar Fractions on *C. elegans* motility. In this bioassay the effect of the 5 Non Polar Fractions (NPFs) of all cannabis varieties tested in this study was tested. The number of full body bends (oscillations) per minute is used as measure for motility after treatment with different varieties from L1 to young adult stage. The concentration tested in this assay was 5 μg/ml in all treatment group samples, resulting in an a cannabinoid concentration that matches with the cannabinoid concentration in the Polar Fractions (PFs) as shown in Figure 4. No significant effect could be observed in the motility after exposure of *C. elegans* to the NPFs of the different varieties. One-way ANOVAs were used to compare the vehicle control conditions Dimethyl sulfoxide (DMSO) to the experimental conditions. MilliQ water (MQ) is the water control. Green bars denote: motility of *C. elegans* exposed to NPFs of different cannabis varieties; brown bars denote: vehicles.

**Additional file 5: Additional file S5.** Effect of Non Polar Fractions on the avoidance of a noxious smell. The effect of Non Polar Fractions (NPFs_ of all varieties selected in this study was tested on avoidance of a noxious smell The time until one full reversal backwards movement was scored after exposure of NPF treated nematodes with 1-octanol. Nematodes have been treated with NPF extracts of different varieties from L1 to young adult stage. The concentration tested in this assay was 5 μg/ml resulting in an a cannabinoid concentration that matches with the cannabinoid concentration in the Polar Fractions (PFs) as shown in Figure 5. Interestingly, the NPFs from cultivars MGC 1003, MGC 1007, MGC 1009, MGC 1013, MGC 1027, MGC 1046, and MGC 1101 had no significant effect on the avoidance of a noxious smell in in contrast to the PFs of the same cultivars. However, the NPFs of extracts of Cannabidiol (CBD)-rich varieties MGC 1074 and MGC 1122 showed a strong significant effect, indicating that also compounds present in a non-polar cannabis fraction (e.g. cannabinoids or terpenoids) show health-related effects in the *C. elegans* test system. One-way ANOVAs were used to compare the vehicle control conditions Dimethyl sulfoxide (DMSO) to the experimental conditions. Milli Q water (MQ) represents the water control. Green bars denote: the effect on avoidance by *C. elegans* exposed to NPFs of different cannabis varieties; brown bars denote: vehicles; *** = False Discovery Rate (FDR)-corrected p < .001.

**Additional file 6: Additional file S6.** Overview of extraction data and cannabinoid content in the Polar Fractions. This table contains and overview of the yields of the Non-Polar Fractions (NPF) and Polar Fractions (PF) after Pressurized Solvent Extraction (PSE) of about 1 gram of dried flowers of each variety tested. The remaining cannabinoid concentration in the PF is given in percentages as the result of the consecutive extraction method used in this study.

**Additional file 7: Additional file S7.** HPLC overlay profile of the pure cannabinoids Δ^9^-tetrahydrocannabinol (THC), Δ^9^-tetrahydrocannabinol-acid (THCA) and cannabidiol (CBD). HPLC analysis was performed by injecting each purified cannabinoid separately.

**Additional file 8: Additional file S8.** The effect of pure cannabinoids on avoidance of a noxious smell. Pure cannabinoids from different varieties show no effect on *C. elegans* avoidance of a noxious smell. Time until one full reversal backwards movement was scored after nematode exposure to 1-octanol in animals treated with 0.0143 ug/mL cannabidiol (CBD) (similar to the concentration in the Polar Fraction (PF) of MGC 1101), pure cannabinoids containing 0.0125 ug/mL CBD (similar to the concentration in the PF of MGC 1074), pure cannabinoids contain 0.0262 ug/mL Δ^9^-tetrahydrocannabinol-acid (THCA) & 0.0133 ug/mL Δ^9^-tetrahydrocannabinol (THC) (similar to the concentration in the PF of MGC 1101), and pure cannabinoids contain 0.0278 ug/mL THCA & 0.0140 ug/mL THC (similar to the concentration in the PF of MGC 1106). A one-way ANOVA was used to compare the vehicle control conditions ethanol to the experimental conditions. Milli Q (MQ) represents the water control group. Green bars denote motility of *C. elegans* exposed to CBD and THCA/THC of different cannabis varieties; brown bars denote controls.

**Additional file 9: Additional file S9.** The effect of pure cannabinoids on *C. elegans* avoidance of a noxious smell with the presence (green bars) or absence (brown bars) of added dopamine. No significant effect can be observed. MilliQ water (MQ) represents the water treated control group. Effects of nematode on the avoidance of 1-octanol in the presence (green bars) or absence (brown bars) of added dopamine. Nematodes were treated from L1 to young adult stage with either 0.0143 ug/mL cannabidiol (CBD) (similar to the concentration in the Polar Fraction (PF) of MGC 1101), pure cannabinoids containing 0.0125 ug/mL CBD (similar to the concentration in the PF of MGC 1074), pure cannabinoids contain 0.0262 ug/mL Δ^9^-tetrahydrocannabinol-acid (THCA) & 0.0133 ug/mL Δ^9^-tetrahydrocannabinol (THC) (similar to the concentration in the PF of MGC 1101), and pure cannabinoids contain 0.0278 ug/mL THCA & 0.0140 ug/mL THC (similar to the concentration in the PF of MGC 1106). A two-way ANOVA was used to compare the vehicle control conditions ethanol to the experimental conditions in the presence or absence of added dopamine.

**Additional file 10: Additional file S10.** Overview of the selected cannabis varieties used in this study and the effects as based on 25 years of human experiences. The effects were reported by surveys among 200 patients in the Netherlands using medicinal cannabis from MariPharms medicinal-cannabis breeding program. Varieties in green columns: Δ^9^ -tetrahydrocannabinol (THC)-rich; varieties in brown columns: cannabidiol (CBD)-rich varieties. √ indicates an reported effect, blank wells indicates an effect not reported by the patients.

## Data Availability

The datasets supporting the conclusions of this article are included within the article and its additional files.

## Abbreviations

MGC: Medical-grade cannabis
PF: Polar fraction
NPF: Non-polar fraction
NPF_dec_: Non-polar fraction after decarboxylation
*C. elegans*: *Caenorhabditis elegans*
THC: Δ^9^-tetrahydrocannabinol
CBD: Cannabidiol
^1^H-NMR: Proton-nuclear-magnetic-resonance
FDR: False discovery rate
MQ: Milli-Q water
