# Supplementary figures and images for: C. elegans as a test system to study relevant compounds that contribute to the specific health-related effects of different cannabis varieties
Source: J Cannabis Res. 2022 Oct 3;4:53. doi: 10.1186/s42238-022-00162-9 (PMC9528106; doi:10.1186/s42238-022-00162-9)

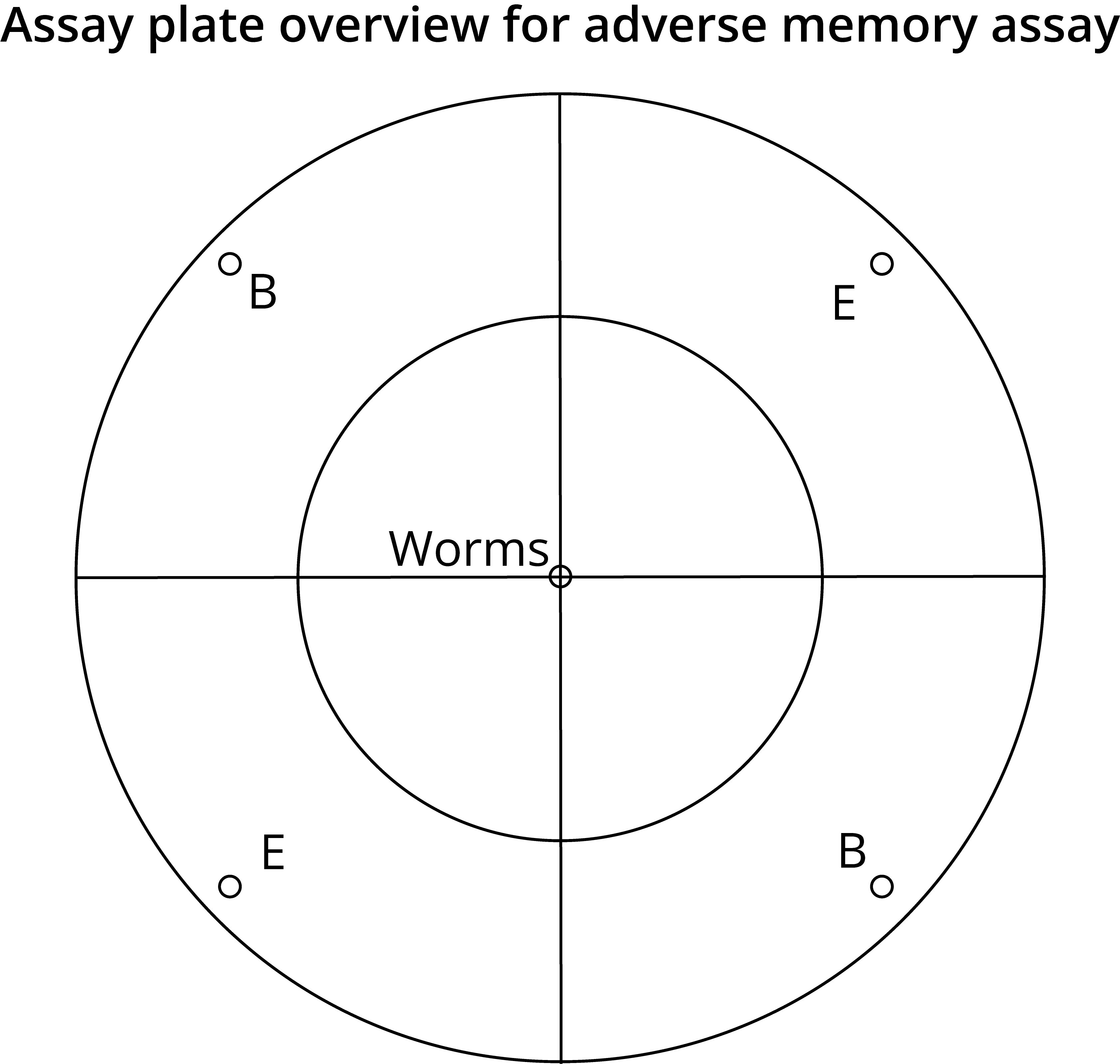

Supplement: Supplementary file 1 — Additional file 1: Additional file S1. Schematic overview of the agar assay plate used for the adverse memory assay. After treatment with cannabis extracts from L1 stage to young adulthood, nematodes were starved in the presence or absence of butanol (see material and methods). After a starvation period, nematodes were allowed to recover for several hours and then assessed for avoidance behavior towards butanol. Nematodes were placed in the middle of the plate and the amount of nematodes at B and E was scored after one hour. Quadrants E were immersed with sodium azide combined with 1% ethanol and quadrants B were immersed with sodium azide in combination with 1% butanone. Nematodes were pipetted in the center of the plate and the movements to the different quadrants after treatment with cannabis extracts was monitored. [file 42238_2022_162_MOESM1_ESM.tif]

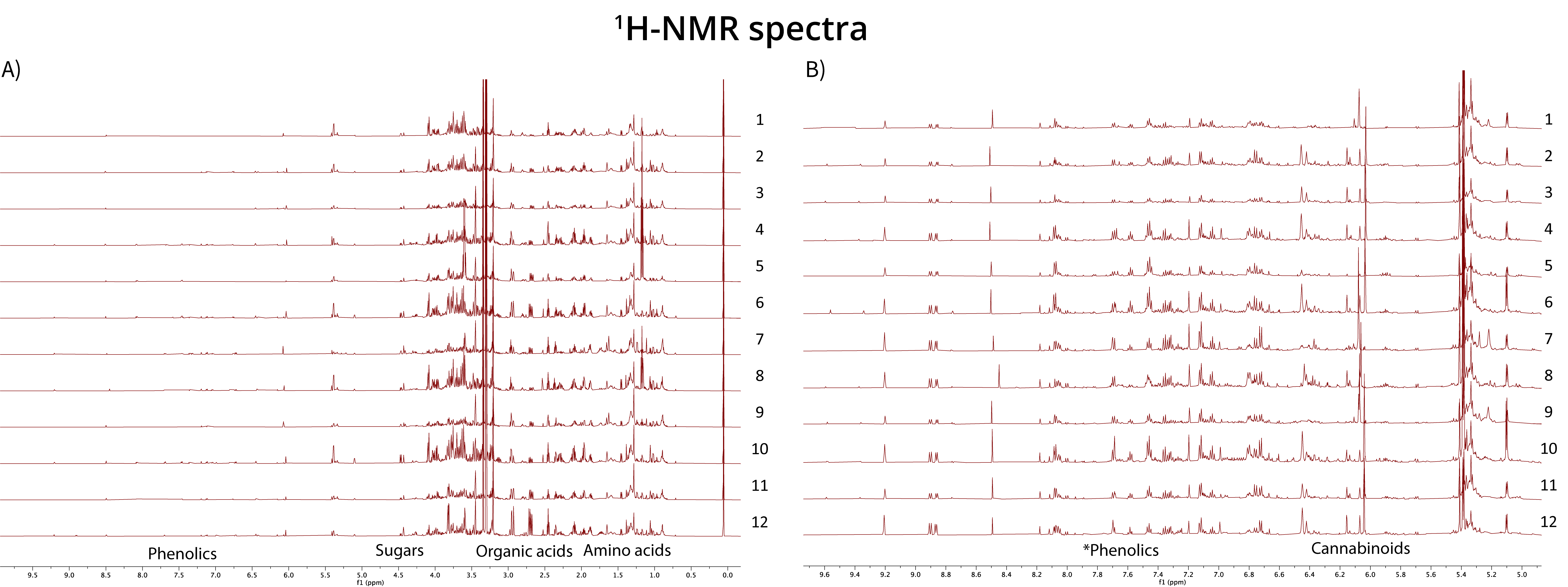

Supplement: Supplementary file 2 — Additional file 2: Additional file S2.1H-NMR spectra of the 12 different genotypes. (A) Full range of 1H-NMR spectra. Number indicates the different genotypes. The region (10.0-5.0 ppm) was expanded (B). 1: MGC 1046, 2: MGC 1010, 3: MGC 1007, 4: MGC 1074, 5: MGC 1027, 6: MGC 1122, 7: MGC 1004, 8: MGC 1009, 9: MGC 1003, 10: MGC 1006, 11: MGC 1001, 12: MGC 1013. The region where the metabolites typically appeared are mentioned in the figure such as amino acids, sugars and cannabinoids. *Phenolics indicates compounds containing a phenolic ring in the molecules. [file 42238_2022_162_MOESM2_ESM.tif]

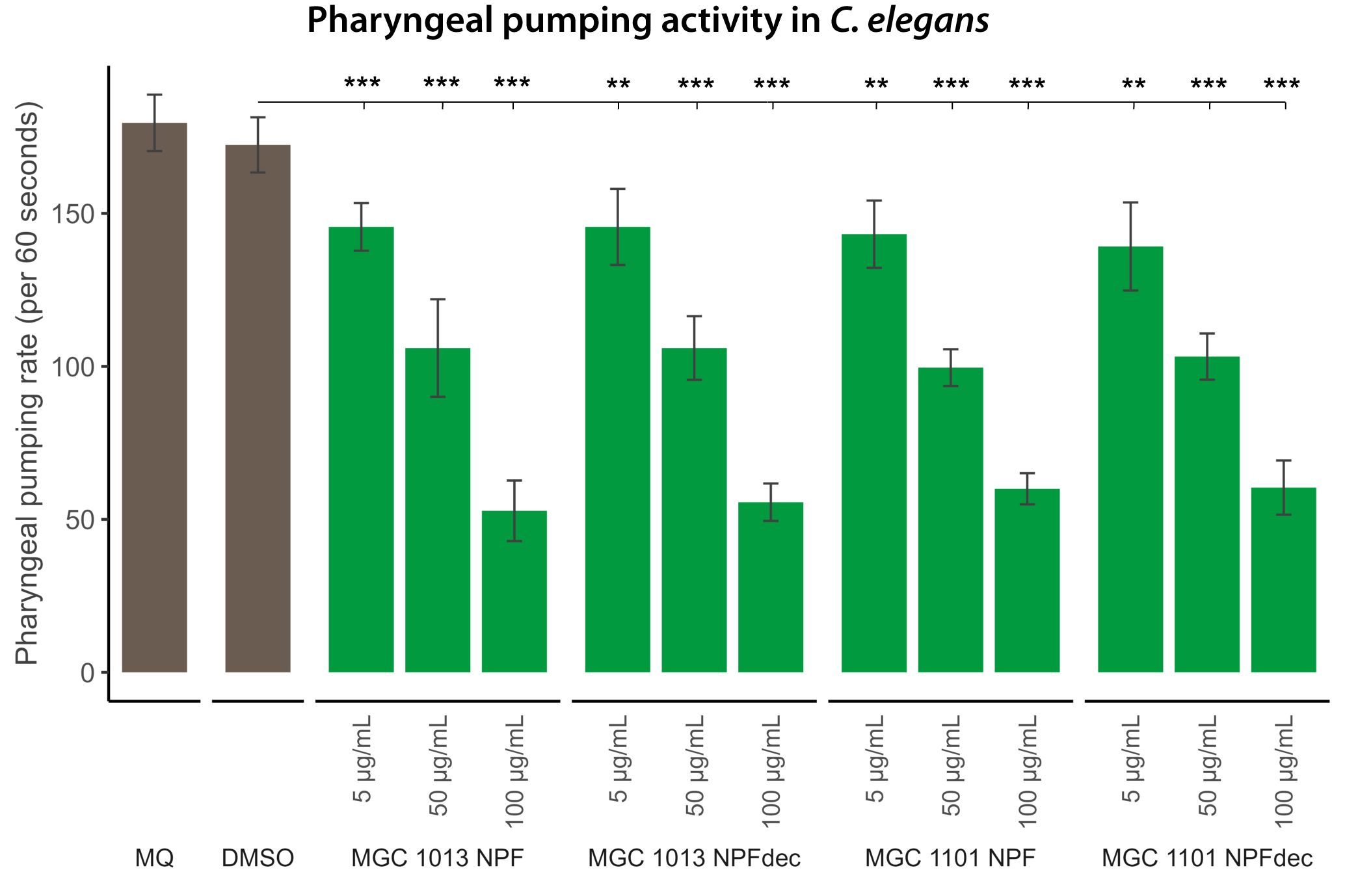

Supplement: Supplementary file 3 — Additional file 3: Additional file S3. Pharyngeal pumping activity of the Non-Polar Fractions (NPF) of the cultivars MGC 1013 and MGC 1101. In this assay C. elegans was exposed to different concentrations (5, 50 and 100 μg/mL) of the Non-Polar Fractions both in carboxylated (NPFdec) and non-decarboxylated (NPF) form. Nematodes were treated from L1 stage to young adulthood with NPF. Pharyngeal pumping frequency is plotted on the Y axis. Pharyngeal pumping activity of all fractions was decreased possibly due to toxicity (see Figure 1). The cannabinoid concentration in the 5 μg/ml NPF of MGC 1013 and MGC 1101 matches with the cannabinoid concentration in the 100 μg/ml PF fractions of MGC 1013 and MGC 1101 respectively (Figure 3). A one-way ANOVA and post-hoc Welch tests were used to compare the vehicle control condition Dimethyl sulfoxide (DMSO) to the experimental conditions. *** = False Discovery Rate (FDR)-corrected p < .001; ** = FDR-corrected p < .01. [file 42238_2022_162_MOESM3_ESM.tif]

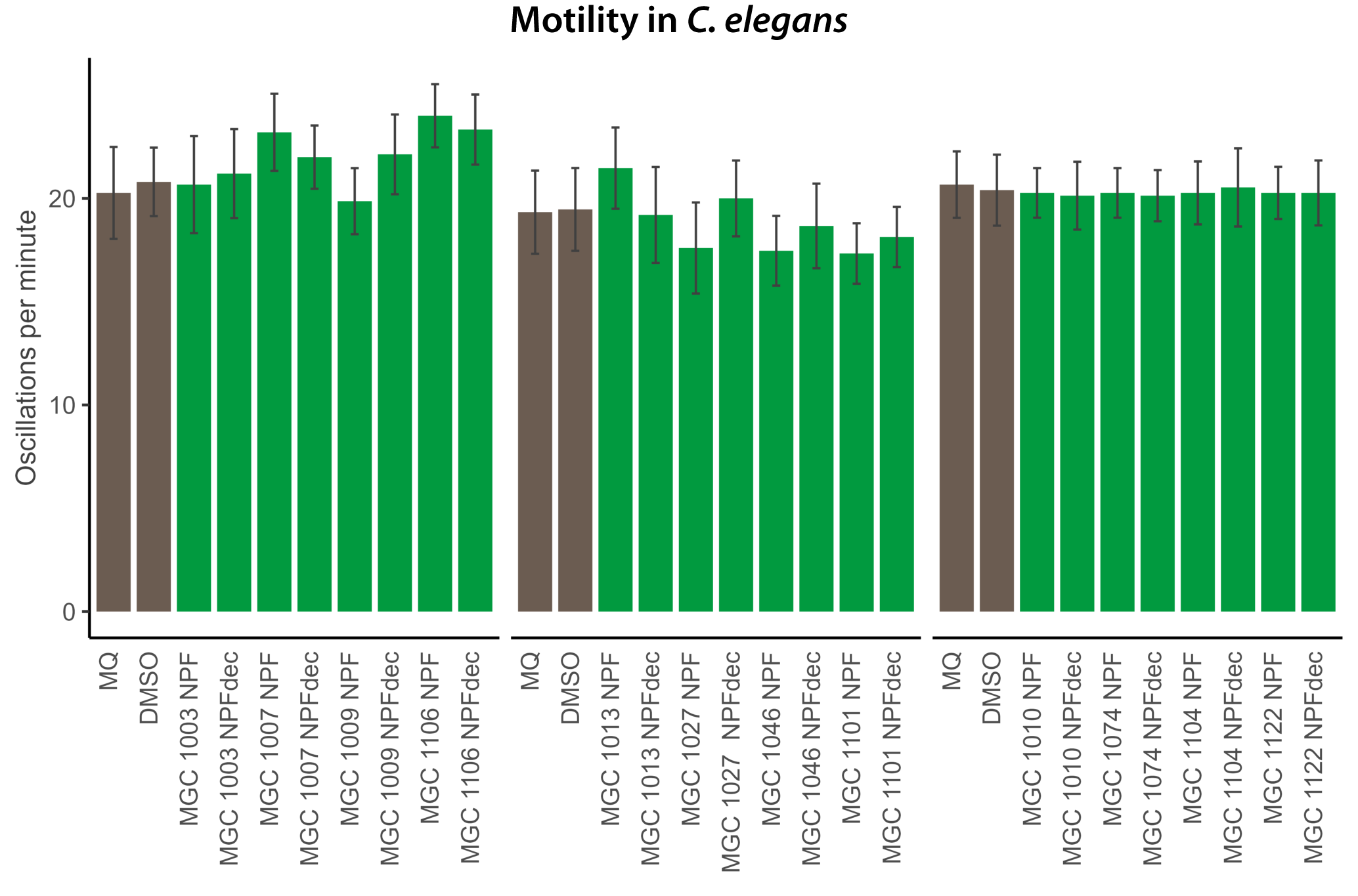

Supplement: Supplementary file 4 — Additional file 4: Additional file S4. Effect of Non Polar Fractions on C. elegans motility. In this bioassay the effect of the 5 Non Polar Fractions (NPFs) of all cannabis varieties tested in this study was tested. The number of full body bends (oscillations) per minute is used as measure for motility after treatment with different varieties from L1 to young adult stage. The concentration tested in this assay was 5 μg/ml in all treatment group samples, resulting in an a cannabinoid concentration that matches with the cannabinoid concentration in the Polar Fractions (PFs) as shown in Figure 4. No significant effect could be observed in the motility after exposure of C. elegans to the NPFs of the different varieties. One-way ANOVAs were used to compare the vehicle control conditions Dimethyl sulfoxide (DMSO) to the experimental conditions. MilliQ water (MQ) is the water control. Green bars denote: motility of C. elegans exposed to NPFs of different cannabis varieties; brown bars denote: vehicles. [file 42238_2022_162_MOESM4_ESM.tif]

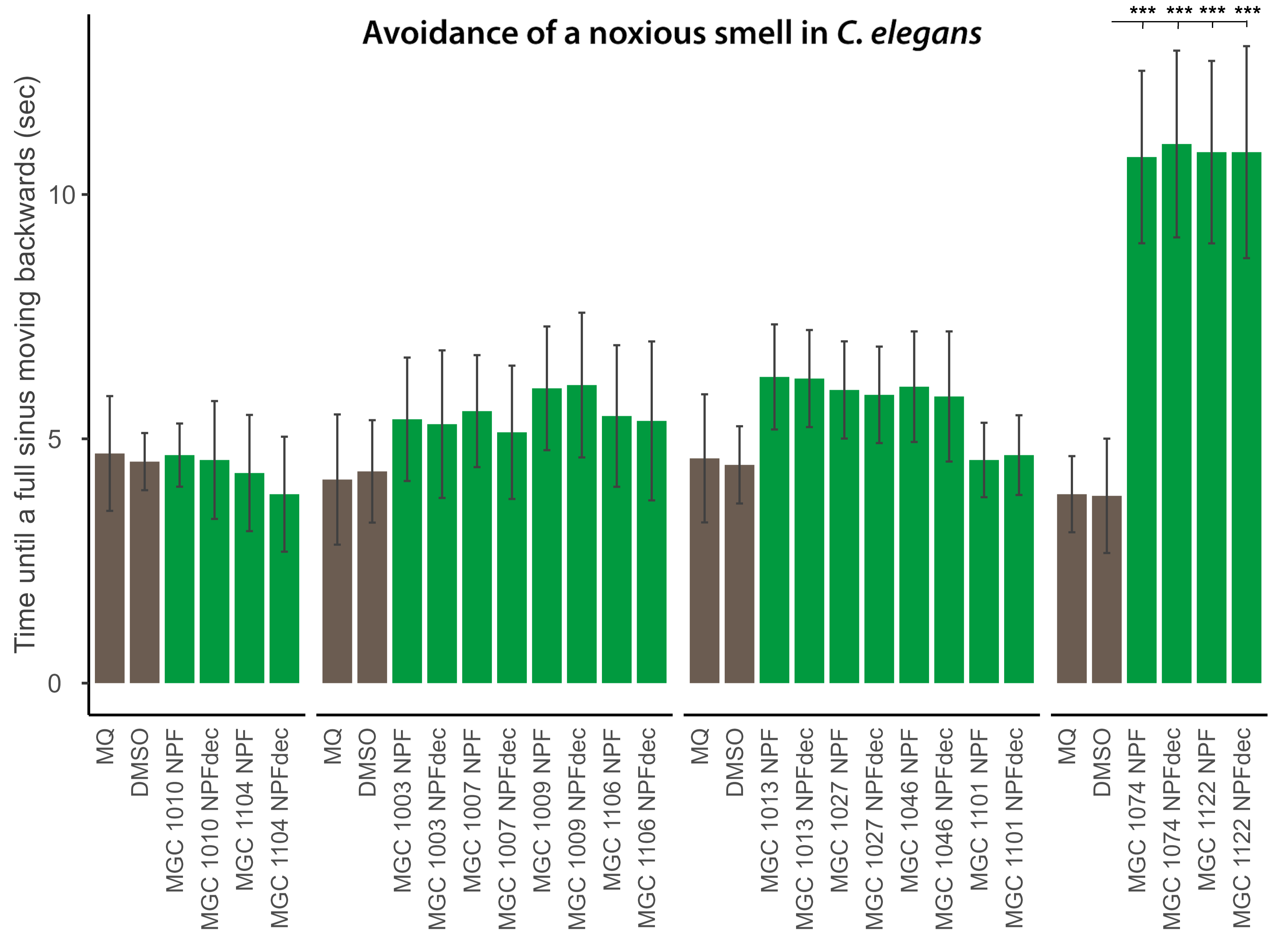

Supplement: Supplementary file 5 — Additional file 5: Additional file S5. Effect of Non Polar Fractions on the avoidance of a noxious smell. The effect of Non Polar Fractions (NPFs_ of all varieties selected in this study was tested on avoidance of a noxious smell The time until one full reversal backwards movement was scored after exposure of NPF treated nematodes with 1-octanol. Nematodes have been treated with NPF extracts of different varieties from L1 to young adult stage. The concentration tested in this assay was 5 μg/ml resulting in an a cannabinoid concentration that matches with the cannabinoid concentration in the Polar Fractions (PFs) as shown in Figure 5. Interestingly, the NPFs from cultivars MGC 1003, MGC 1007, MGC 1009, MGC 1013, MGC 1027, MGC 1046, and MGC 1101 had no significant effect on the avoidance of a noxious smell in in contrast to the PFs of the same cultivars. However, the NPFs of extracts of Cannabidiol (CBD)-rich varieties MGC 1074 and MGC 1122 showed a strong significant effect, indicating that also compounds present in a non-polar cannabis fraction (e.g. cannabinoids or terpenoids) show health-related effects in the C. elegans test system. One-way ANOVAs were used to compare the vehicle control conditions Dimethyl sulfoxide (DMSO) to the experimental conditions. Milli Q water (MQ) represents the water control. Green bars denote: the effect on avoidance by C. elegans exposed to NPFs of different cannabis varieties; brown bars denote: vehicles; *** = False Discovery Rate (FDR)-corrected p < .001. [file 42238_2022_162_MOESM5_ESM.tiff]

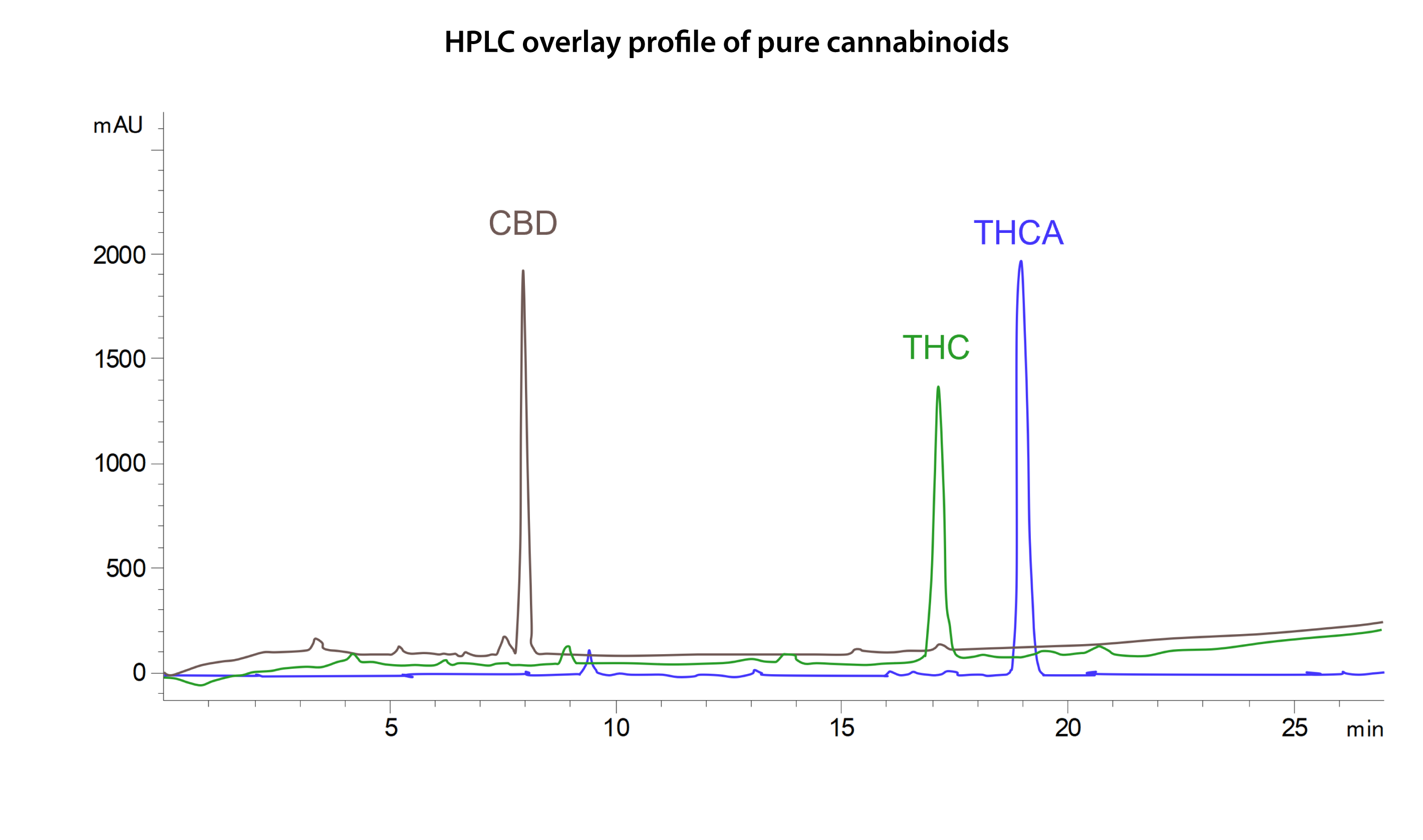

Supplement: Supplementary file 7 — Additional file 7: Additional file S7. HPLC overlay profile of the pure cannabinoids Δ9-tetrahydrocannabinol (THC), Δ9-tetrahydrocannabinol-acid (THCA) and cannabidiol (CBD). HPLC analysis was performed by injecting each purified cannabinoid separately. [file 42238_2022_162_MOESM7_ESM.tif]

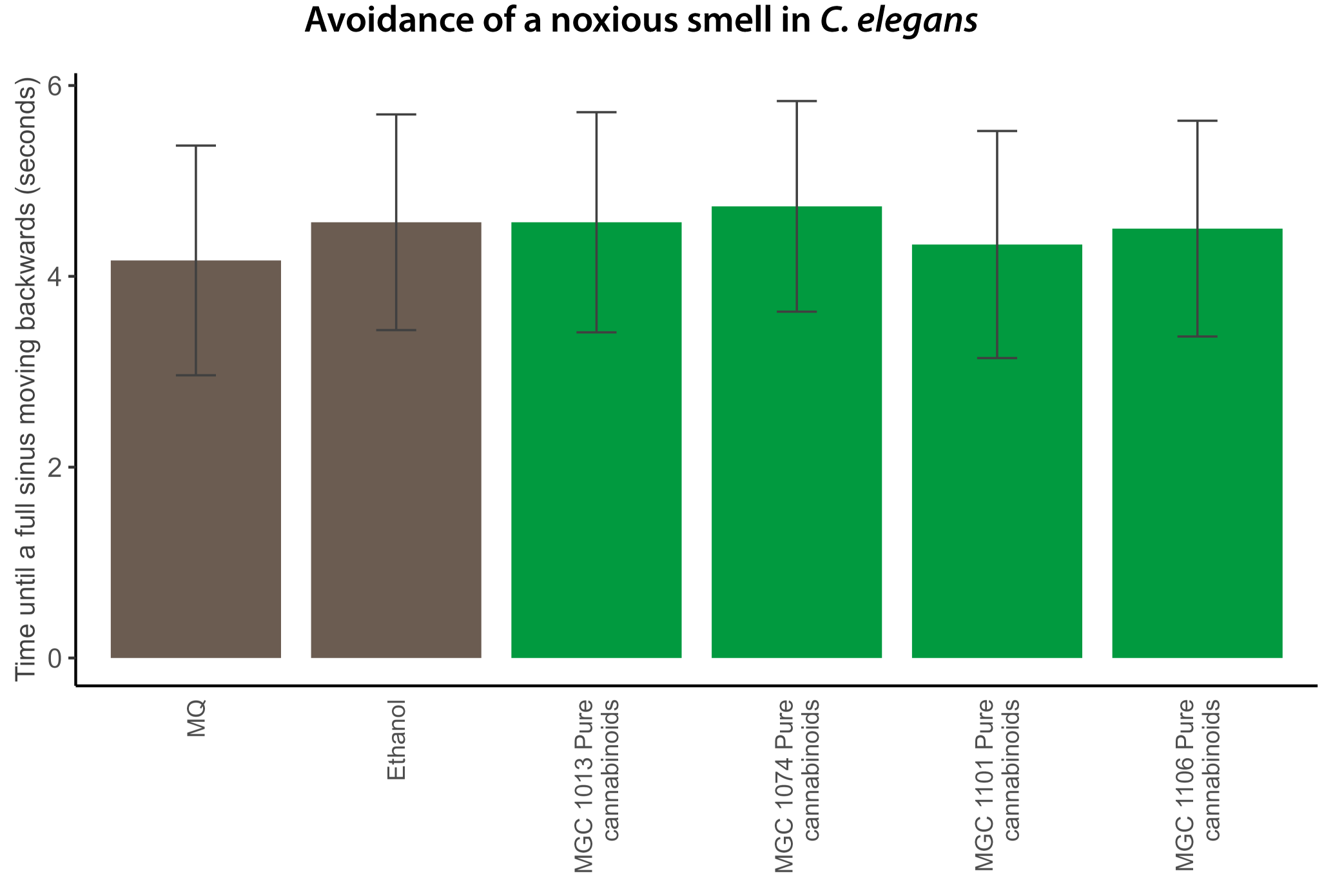

Supplement: Supplementary file 8 — Additional file 8: Additional file S8. The effect of pure cannabinoids on avoidance of a noxious smell. Pure cannabinoids from different varieties show no effect on C. elegans avoidance of a noxious smell. Time until one full reversal backwards movement was scored after nematode exposure to 1-octanol in animals treated with 0.0143 ug/mL cannabidiol (CBD) (similar to the concentration in the Polar Fraction (PF) of MGC 1101), pure cannabinoids containing 0.0125 ug/mL CBD (similar to the concentration in the PF of MGC 1074), pure cannabinoids contain 0.0262 ug/mL Δ9-tetrahydrocannabinol-acid (THCA) & 0.0133 ug/mL Δ9-tetrahydrocannabinol (THC) (similar to the concentration in the PF of MGC 1101), and pure cannabinoids contain 0.0278 ug/mL THCA & 0.0140 ug/mL THC (similar to the concentration in the PF of MGC 1106). A one-way ANOVA was used to compare the vehicle control conditions ethanol to the experimental conditions. Milli Q (MQ) represents the water control group. Green bars denote motility of C. elegans exposed to CBD and THCA/THC of different cannabis varieties; brown bars denote controls. [file 42238_2022_162_MOESM8_ESM.tif]

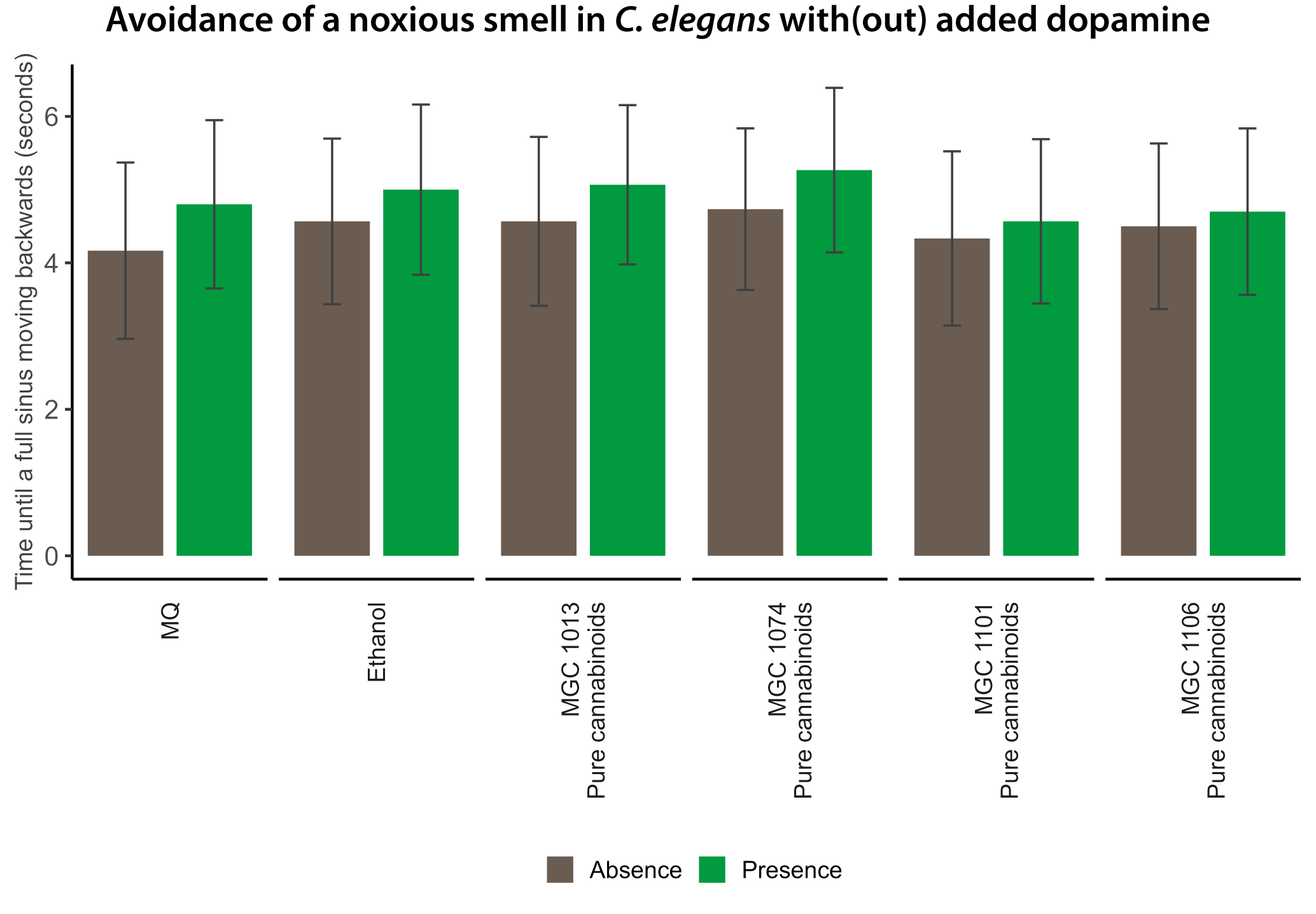

Supplement: Supplementary file 9 — Additional file 9: Additional file S9. The effect of pure cannabinoids on C. elegans avoidance of a noxious smell with the presence (green bars) or absence (brown bars) of added dopamine. No significant effect can be observed. MilliQ water (MQ) represents the water treated control group. Effects of nematode on the avoidance of 1-octanol in the presence (green bars) or absence (brown bars) of added dopamine. Nematodes were treated from L1 to young adult stage with either 0.0143 ug/mL cannabidiol (CBD) (similar to the concentration in the Polar Fraction (PF) of MGC 1101), pure cannabinoids containing 0.0125 ug/mL CBD (similar to the concentration in the PF of MGC 1074), pure cannabinoids contain 0.0262 ug/mL Δ9-tetrahydrocannabinol-acid (THCA) & 0.0133 ug/mL Δ9-tetrahydrocannabinol (THC) (similar to the concentration in the PF of MGC 1101), and pure cannabinoids contain 0.0278 ug/mL THCA & 0.0140 ug/mL THC (similar to the concentration in the PF of MGC 1106). A two-way ANOVA was used to compare the vehicle control conditions ethanol to the experimental conditions in the presence or absence of added dopamine. [file 42238_2022_162_MOESM9_ESM.tif]
