# Supplementary material for: C. elegans as a test system to study relevant compounds that contribute to the specific health-related effects of different cannabis varieties
Source: J Cannabis Res. 2022 Oct 3;4:53. doi: 10.1186/s42238-022-00162-9 (PMC9528106; doi:10.1186/s42238-022-00162-9)
Supplement: Supplementary file 6 — Additional file 6: Additional file S6. Overview of extraction data and cannabinoid content in the Polar Fractions. This table contains and overview of the yields of the Non-Polar Fractions (NPF) and Polar Fractions (PF) after Pressurized Solvent Extraction (PSE) of about 1 gram of dried flowers of each variety tested. The remaining cannabinoid concentration in the PF is given in percentages as the result of the consecutive extraction method used in this study. [file 42238_2022_162_MOESM6_ESM.docx]

Extraction data and cannabinoid content in the Polar Fractions (PF).

| Variety | Extracted with PSE (g) | Yield NPF (mg) | Yield PF (mg) | Cannabinoid content in PF (%) | | |
| --- | --- | --- | --- | --- | --- | --- |
|  |  |  |  | THCA | THC | CBD |
| MGC 1003  MGC 1007  MGC 1009  MGC 1010  MGC 1013  MGC 1027  MGC 1046  MGC 1074  MGC 1101  MGC 1104  MGC 1106  MGC 1122 | 1.00  1.09  0.89  0.98  0.92  0.98  0.94  1.07  1.01  0.90  0.96  0.96 | 257  222  115  67  99  163  152  153  175  194  157  259 | 168  170  175  149  168  215  158  209  159  247  179  191 | 0.039  0.032  0.014  0.017  0.000  0.041  0.039  0.000  0.026  0.042  0.028  0.000 | 0.013  0.007  0.003  0.008  0.000  0.019  0.017  0.000  0.013  0.016  0.014  0.000 | 0.000  0.000  0.000  0.000  0.014  0.000  0.000  0.012  0.000  0.000  0.000  0.040 |
