# Supplementary material for: C. elegans as a test system to study relevant compounds that contribute to the specific health-related effects of different cannabis varieties
Source: J Cannabis Res. 2022 Oct 3;4:53. doi: 10.1186/s42238-022-00162-9 (PMC9528106; doi:10.1186/s42238-022-00162-9)
Supplement: Supplementary file 10 — Additional file 10: Additional file S10. Overview of the selected cannabis varieties used in this study and the effects as based on 25 years of human experiences. The effects were reported by surveys among 200 patients in the Netherlands using medicinal cannabis from MariPharms medicinal-cannabis breeding program. Varieties in green columns: Δ9 -tetrahydrocannabinol (THC)-rich; varieties in brown columns: cannabidiol (CBD)-rich varieties. √ indicates an reported effect, blank wells indicates an effect not reported by the patients. [file 42238_2022_162_MOESM10_ESM.docx]

Cannabis varieties effects based on 25 years of human experiences.

|  | **MGC 1003** | **MGC 1007** | **MGC 1009** | **MGC 1010** | **MGC 1013** | **MGC 1027** | **MGC 1046** | **MGC 1074** | **MGC 1101** | **MGC 1104** | **MGC 1106** | **MGC 1122** |
| --- | --- | --- | --- | --- | --- | --- | --- | --- | --- | --- | --- | --- |
| ***Anti-addiction*** |  |  |  |  | **√** |  |  |  |  |  |  |  |
| ***Stimulates focus/concentration*** |  |  |  |  | **√** |  |  | **√** |  |  |  |  |
| ***Relaxes the mind*** | **√** |  |  |  | **√** |  |  |  |  |  |  | **√** |
| ***Relaxes the body*** | **√** | **√** | **√** |  |  | **√** | **√** |  |  |  |  |  |
| ***Inhibits appetite*** |  |  |  |  | **√** |  |  |  |  |  |  |  |
| ***Stimulates appetite*** |  |  |  |  |  |  |  |  | **√** |  |  |  |
| ***Inhibits nausea*** |  |  |  |  |  |  |  |  | **√** |  |  |  |
| ***Reduces pain*** |  |  |  |  |  |  |  |  |  | **√** |  |  |
| ***Stimulates happiness/euphoria*** |  |  | **√** | **√** |  |  |  |  |  | **√** |  |  |
| ***Provides energy*** |  |  |  |  |  |  |  |  |  |  | **√** |  |
| ***Stimulates creativity*** |  |  |  |  |  |  |  |  |  |  | **√** |  |
| ***Stimulates libido*** |  |  |  |  |  |  |  |  |  |  | **√** |  |
| ***Helps sleeping*** |  | **√** | **√** |  |  | **√** |  |  |  |  |  |  |
| ***Inhibits thinking*** |  | **√** | **√** |  |  |  |  |  |  |  |  |  |
